# Abstracts of Papers Presented at the Connective Tissue Oncology Society 4th Annual Scientific Meeting, 12–14 November 1998, Vancouver, BC, Canada

**DOI:** 10.1080/13577149977866

**Published:** 1999-03


*Sarcoma* (1999) **3**, 43–61

### Abstracts of Papers Presented at the Connective Tissue Oncology Society 4th Annual Scientific Meeting, 12–14 November 1998, Vancouver, BC, Canada

**Scientific Program Committee Members:**

Brian O'Sullivan, Canada (Chairman)
Colin Cooper, UK (Basic Science Program Chairman)

Alberto Azzarelli, Italy
Nicola Baldini, Italy
Robert Bell, Canada
Robert Benjamin, USA
Sybil Biermann, USA
Vivien Bramwell, Canada
Lee Helman, USA
Jonathan Lewis, USA
Robert Marcus, USA
Ole Nielsen, Denmark
David Panicek, USA
Piero Picci, Italy
Martin Robinson, UK
Ira Spiro, USA
Frits van Coevorden, The Netherlands
Jaap Verweij, The Netherlands
Sharon Weiss, USA

1357-714X/99/010043-19 $9.00 © 1999 Taylor & Francis Ltd

### RANDOMIZED STUDY COMPARING DOXORUBICIN (D) TO TAXOTERE™ (T) IN PREVIOUSLY UNTREATED SOFT TISSUE SARCOMAS (STS)

*J. Verweij, I. Judson, M. Lee, W. Ruka, J. Buesa, R. Coleman, E. di Paola, D. Locci-Tonelli, M. van Glabbeke, T. Tursz*

*The EORTC Soft Tissue and Bone Sarcoma Group (STBSG), 1200 Brussels, Belgium*

STBSG previously reported activity of T in 2nd line chemotherapy. In order to establish the first line activity of T in STS we performed a randomized study comparing to D given as a dose of 75 mg/m$^2$ iv bolus every 3 weeks to T given at a dose of 100 mg/m$^2$ as 1-hr infusion every 3 weeks with corticosteroid comedication, in chemotherapy-nonpretreated patients (pts). At progression, cross-over to the other treatment arm was allowed. Eighty-six pts (43 on D, 43 on T) were entered on study. Median age was 52 years (range 20–76), median WHO PS 1 (range 0–2), 60% were males, 34% of tumors were leiomyosarcomas and 50% of pts had lung metastases. At present 81 pts are fully evaluable. The median nr. of courses received was 3 for D (range 1–7) and 2 for T (range 1–7). Achieved dose intensity (DI) over all cycles was 98% from projected DI for D and 99% for T. On a per patient basis the median granulocyte nadir was 0.6 (range 0.0–5.3 × 10$^9$/l) for T and 0.5 (range 0.1–8.4) for D, the febrile neutropenia rate being 12% and 22% respectively.

D was more emetogenic (75 vs 27%) and induced more stomatitis (64 vs 47%, mainly grade 1/2), while T induced more diarrhea (59 vs 37%) and edema (37 vs 22%, mainly mild to moderate). Objective partial responses were seen in 13 pts (30%, 95%, CI: 17–46%) on D and none on T. Stable disease was seen in 50% of pts on D and 44% on T. Median TTP was 6 months for D and 2 months for T (p=0.02).

Thirty-two patients crossed-over to the other study arm after previous failure. There were 2 PRs (13%) in 16 pts on second line D and no responses in 16 pts on second line T.

While the activity of D was once again confirmed, T appears to be inactive in STS.

### EPIDEMIOLOGIC ASPECTS OF SOFT TISSUE SARCOMAS (STS)—CONSEQUENCES FOR FUTURE CLINICAL STS-TRIALS

*P. Nijhuis, M. Schaapveld, R. Otter, H. Hoekstra*

*Dept. of Surgical Oncology, Groningen University Hospital, and the CCCN, PO Box 30.001, 9700 RB, Groningen, The Netherlands*

*Introduction*: Only limited valid data regarding epidemiologic and treatment-related aspects of STS are available. The purpose of this study was to gain insight into these aspects, based on the population-based cancer registry of the Comprehensive Cancer Center North-Netherlands (CCCN).

*Methods*: The CCCN Cancer Registry is based on data of all yearly diagnosed cancer pts in the 19 hospitals, covering an area of 2.1 million people. All registered, 391 primary STS, with exception of urogenital and gastrointestinal STS, diagnosed between 1989–95 were analyzed with respect to incidence, sex and age distribution, anatomical site, histology, tumor size, initial metastases, and treatment performed.

*Results*: *Incidence STS*: 3/100.000/yr. *Sex ratio*: 205♂:186♀. *Age distribution*: 0–19 yrs: 7%, 20–69 yrs: 58%, 70+yrs: 35%. *Primary site*: trunk 31%, lower limb 29%, upper limb 15%, head/neck 14.5%, retroperit. 9%, nos 1.5%. *Histology*: MFH 21%, liposarc. 21%, leiomyosarc. 18%, dermatofibrosarc. 17%, fibrosarc. 8%, rhabdomyosarc. 6%, nos 9%. Histological distribution equal in both sexes. *Other analysis-based data*:

| | | |
|---|---|---|
| *Sex-incidence* | ♂ 3.4/100.000/yr; ♀ 2.7/100.000/yr | NS |
| *Age-incidence* | 0–19 yrs: 0.8; 20–69 yrs: 2.9; 70+: 11.8 | P<.001 |
| *Primary site* | ♂: arms and head/neck; ♀: legs | P=.04 |
| *Histology (i)* | leiomyosarcomas in retroperitoneum; liposarcomas in retroperit. and legs | P<.001 |
| *Histology (ii)* | <10 yrs: rhabdoymosarcomas; 70+: fibro- & leiomyosarcomas | P<.001 |
| *Tumor size* | head/neck: T1; retroperitoneum: T2 | P<.001 |

Incidence lymph node metastases 2.1%, distant metastases 2.3%. *Treatment*: 318 pts curative intent (81%), 41 pts palliative (11%), 32 pts no treatment (8%); older pts received more often palliative or no treatment at all.

*Conclusion*: Incidence of STS is significantly increasing with age, whereas, in the meantime, older pts more often receive no treatment. It is therefore expected that a substantial proportion of pts with STS cannot be treated in scientific studies, since most protocols exclude pts above 65 years. Slow accrual for such studies is therefore expected. Most patients are treated with curative intent (81%). Initial lymph node or distant metastases are rare (2%).

### EPIRUBICIN IS NOT A SUBSTITUTE FOR DOXORUBICIN IN ADVANCED SOFT TISSUE SARCOMAS. FINAL DATA FROM THE EORTC SOFT TISSUE AND BONE SARCOMA GROUP

*O. S. Nielsen, P. Dombernowsky, H. Mouridsen, S. Daugaard, M. van Glabbeke, A. Kirkpatrick, J. Verweij*

*EORTC STBSG, EORTC Data Centre, 1200 Brussels, Belgium*

Doxorubicin (dox) still appears to be one of the most active drugs in the treatment of soft tissue sarcomas. However, treatment duration is limited due to cumulative cardiotoxicity. It is therefore important to test anthracycline analogs. At present only few analogs have been evaluated in studies with adequate number of patients. In two studies of the EORTC STBSG we have tested whether epirubicin (epi) is an alternative to standard dose dox in the treatment of chemonaive patients with advanced soft tissue sarcoma. The present report gives the final results of these studies. In the first study 210 patients were randomized to receive either dox or epi both at a dose of 75 mgm$^{-2}$ given as bolus injection at 3-week intervals. Median age was 54 years (18–80 years) and Karnofsky performance score 90 (50–100). No difference in median survival (41 weeks after dox vs 48 weeks after epi) and duration of response (45 weeks after dox vs 77 weeks after epi) was found. Of 167 evaluable patients 36 (22%) had an objective tumor response (10 CR, 26 PR), and the response rate was slightly in favour of dox (25% vs 18%) but at the expense of more toxicity (leucopenia, alopecia, nausea/vomiting). These data indicate that epi may be less toxic than dox when administered in equimolar doses, and could suggest that increasing the epi dose may lead to a greater antineoplastic effect with acceptable toxicity. Moreover, cardiac toxicity may be related to the peak concentration of the drug, and lower toxicity could therefore be expected with fractionated as compared with bolus injections. In view of this, we initiated our second study comparing single-agent standard dose dox with that of two schedules of high dose epi. A total of 334 patients received dox 75 $mgm^{-2}$ day 1, epi 150 $mgm^{-2}$ day 1 or epi 50 $mgm^{-2}$ $day^{-1}$, days 1, 2 and 3, all given as bolus injection at 3 week intervals. Median age was 52 years (19–70 years) and WHO performance score 1 (0–2). Of 314 evaluable patients 45 (14%) had an objective tumor response (8 CR, 35 PR). There were no differences between the three groups. Median time to progression for the groups dox, epi-150 and epi-50 was 16, 14 and 12 weeks and median survival 45, 47 and 45 weeks, respectively. Neither progression-free nor overall survival differed between the three groups. After the 1st cycle of therapy two patients died of infection and one due to cardiovascular disease, all on epi. Both dose schedules of epi were more myelotoxic than dox. Cardiotoxicity (≥ grade 3) occurred in ≤ 2%. Based on these studies we can conclude, that epi regardless of schedule and dose is not an alternative to dox in the treatment of patients with advanced soft tissue sarcomas. In addition, the results illustrate that the data from small studies of single institutions should always be confirmed by large multi-institutional studies before being taken for granted.

### EWING'S SARCOMA: HYPERFRACTIONATED RADIOTHERAPY WITH CONCURRENT CHEMOTHERAPY: ACUTE TOXICITY. A PROSPECTIVE SINGLE INSTITUTION STUDY

***A. M. Cassoni*, J. S. Whelan**

*The Middlesex Hospital, UCLH, London, W1N 8AA, UK*

Fifty-two patients with EWINGS/PNET were treated with hyperfractionated radiotherapy with planned gaps, concurrent with intensive chemotherapy. Thirty were treated within the EICESS study, eleven in the preceding E2 protocol. All patients requiring radical radiotherapy as part of their initial management were included.

*Patients*: Gender: male 30, female 22. Age (yrs): median 18, range 4–54.

*Site*: Pelvis 19; lower limbs 20; upper limbs & shoulder girdle 6; other 10.

*Chemotherapy*: $IVAD_2$ 16 patients; VAIA 17; EVAIA 14; VIDE 1; CbEC 4.

*Radiotherapy*: adjuvant to surgery, 44.8 Gy/28#/29 days—15 pts; radiotherapy alone, 54.4 Gy/34# in either 3 blocks (46 days)—16 pts or 2 blocks (35 days)—21 pts.

*Results*: *acute skin toxicity*; grade 3 extremities, 5 pts (19%) but in all with perineum in field (14 pts).

*recall reaction*; 2 pts.

*treatment delivery*; radiotherapy—delay of 5–7 days in 3 pts (16%); chemotherapy—omission of anthracycline, planned 5 pts, due to acute toxicity 3 pts, total 15%.

*Conclusion*: the majority of patients can be treated with concurrent chemoradiation without modification of the chemotherapy regimen. Patients with perineum in the field however may need modification of overall radiotherapy time or concurrent drugs. Late toxicity in spinal cord remains a concern.

### CLINICAL ASPECTS OF GIANT LIPOSARCOMAS IN THE RETROPERITONEUM

***Th. van Dalen*, A. N. van Geel, H. J. Hoekstra, F. van Coevorden, A. H. Hennipman**

*Dutch Soft Tissue Sarcoma Group, Servaasbolwerk 14, 3512 NK Utretcht, The Netherlands*

*Background*: Liposarcomatous tumours in the retroperitoneal space are rare. The anatomic localization permits expansion of these neoplasms with symptoms developing only at a late stage of the disease. The resultant giant retroperitoneal masses commonly lead to a defeatist attitude among the treating surgeons. The case histories of patients with "giant" liposarcomas in the retroperitoneal space were studied.

*Methods*: In a retrospective nation-wide study data were collected on 45 patients treated in the Netherlands between 1988 and 1993 for primary giant retroperitoneal liposarcomas. Patients with tumours weighing more than 1 kilogram or measuring more than 10 cm in diameter were included. Median age was 53 years; there were 22 men (49%).

*Results*: Symptoms were longstanding (>6 months) in 1/3 of the patients (16/45), common clinical features were: palpable mass (60%), expanding abdomen (36%) and pain (27%). The intra-abdominal mass was correctly diagnosed as a retroperitoneal sarcoma in 30 patients (67%) prior to definitive treatment. On operation half of the patients (n=22) had tumours in the pararenal space, 8 had pelvical liposarcomas and 7 had central tumours. Median diameter of the tumour was 24 cm. and 29 tumours (64%) were of low-grade malignancy. In 37 patients (82%) radical tumour resection was feasible.

Median follow-up was 6 years, 5-yr. survival for all patients was 59%. Irradical resection (5-yr. surv. 30%), high malignancy grade (5-yr. surv. 25%) and pelvical localisation (5-yr. surv. 38%) were unfavourable prognostic factors. Tumourfree 5-year survival was 30% for all patients. 80% of the patients (25/31) in whom tumour recurred had isolated locoregional recurrences. Following surgery for relapses sarcoma 5-yr. survival was 39%.

*Conclusions*: Despite the large dimensions of "giant" retroperitoneal liposarcomas radical resection is feasible in most patients resulting in reasonable long-term survival. Aggressive surgical therapy is justified in patients with primary retroperitoneal liposarcomas and patients with isolated locoregional relapse.

### STROMAL SARCOMA OF THE STOMACH

***M. Peiper*, T. E. Langwieler, C. Bloechle, C. Zornig**

*Universitäts-Krankenhaus Hamburg-Eppendorg, 20249 Hamburg, Germany*

*Introduction*: Gastric stromal sarcomas (GIST) represent only about 1–3% of all gastric malignancies. Patients generally lack specific symptoms. Despite advances in radiological diagnostics and endoscopy including endosonography, preoperative diagnosis of these tumors is rare. Because of the rarity of these tumors there is a lack in understanding their natural history and the best approach to their treatment. Therefore, we analyzed retrospectively our series of patients with this malignancy.

*Methods*: Twenty patients with GIST underwent surgery at the University-Hospital of Hamburg-Eppendorf from

1979 to 1995. Resection quality was classified according to the UICC. In reviewing the histopathological slides tumor grade (G1–3) was determined according to the grading system for soft tissue sarcomas of Enzinger and Weiss, while classification of tumors were performed according to Fletcher and Franquemont.

*Results*: Symptoms were unspecific. Exact preoperative diagnosis was difficult because of submu-cosal tumor growth and was correctly performed in one patient. Operations varied from excision of the gastric wall to extended gastrectomy (pancreas, spleen, partial liver resection). In 16 cases (80%), tumor could be resected with wide margins (R0). In 4 cases, tumor was found at the resection line (R1). One patient died because of postoperative complications. After a median follow-up of 69 months, 12 patients with R0 resections (including tumors with poor differentiation or infiltration of surrounding organs) lived tumor-free, and two died due to other causes. All four patients with R1 resections died because of tumor disease within 40 months. There was a mean survival time of 59 months and a 5-year-survival rate of 69%.

*Discussion*: Our series shows that not all gastric malignancies have a dismal prognosis, but that gastric stromal sarcomas have a favourable follow-up if resected with wide margins at initial surgery.

### SOFT TISSUE SARCOMA OF THE COLON AND RECTUM

*T. F. Langwieler, C. Zornig, M. Peiper*

*Dept. of Surgery, University Hospital Hamburg-Eppendorf, 20246 Hamburg, Germany*

*Introduction*: Soft tissue sarcoma (STS) of the colon and rectum are rare mesenchymal neoplasm. Consequently, analysis of small series of patients concerning tumor characteristics as well as their treatment and how these factors influence recurrence and patients survival is particularly interesting.

*Methods*: Four consecutive patients with STS of colon and rectum operated on at our institution between 1987 and 1994 were included. In this retrospective study we analysed patient data, surgical therapy, tumor characteristics as well as follow-up.

*Results*: There were 4 patients with a mean age of 49 (range 41–58) years, 100% male. The STS were located in the sigmoid (n=1), rectum (n=2) and colon descendens (n=1). All tumors were resected with wide margins (R0). Two tumors were highly differentiated (G1), 1 moderately (G2) and 1 tumor was grade G3. Postoperative radiation therapy and chemotherapy were administered in the patient with the poorly differentiated tumor. 2 patients developed tumor recurrence: in 1 patient 16 months after complete resection peritoneal metastasis without local recurrence occurred while the second patient developed liver metastasis 9 months after complete tumor resection. Two patients died after 18 and 29 months, respectively, due to metastatic tumor disease. The other two patients are without evidence of disease after 45 and 61 months.

*Conclusion*: The prognosis of sarcomas of colon and rectum is poor. Complete surgical excision is the optimal therapy. Nevertheless, adjuvant therapy might be favourable and we therefore consider this treatment in the future even for patients with tumor resection.

### PROGNOSTIC FACTORS IN RETROPERITONAL SARCOMA. ANALYSIS OF A SERIES OF 165 PATIENTS OF THE FRENCH CANCER CENTER FEDERATION (FNCLCC) SARCOMA GROUP

*E. Stöckle*, F. X. Sastre, S. Bonvalot, G. Depadt, P. Terrier, J. Cuisenier, J. M. Coindre*, N. B. Bui**

**Institut Bergonié, 33076 Bordeaux Cedex, France*

*Objective*: To analyse prognostic factors in a large multicentric series of retroperitoneal sarcoma.

*Methods*: A series of 165 patients (pts) with primary retroperitoneal sarcoma (retroSTS) registered in the FNCLCC sarcoma group data was analyzed. Pts referred for tumors recurrences were not considered. Median age was 54 years (16–82) and the sex-ratio was 0.9. The median follow-up was 47 months. In only 6% of cases, the tumor was <5 cm; 31% showed evidence of vasculonervous involvement; 5% were N+ and 12% were M+ at referral. MFH, liposarcoma and leiomyosarcoma represented 66% of histological types; tumor grade was: G1 17%, G2 40%, G3 43%. 150 pts had surgery, with complete tumor removal in 65%. Radiotherapy was given to 92 pts and chemotherapy to 77 pts. At the end of first treatment, 118 pts (72%) were considered as free of disease. Univariate and multivariate analysis of prognostic factors for Complete Remission (CR), Overall Survival (OS), Local Recurrence Free Interval (LRFI), Metastatic Free Interval (MFI) and Disease Free Interval (DFI) were done.

*Results*: 5-years OS was 45.7% and 5-year DFI was 28%, with a 5-year LRFI of 31% and MFI of 66%. A disease-free status at completion of treatment was correlated with metastases at entry, tumor grade and vasculonervous involvement. At multivariate analysis of the entire group, OS was independently correlated with grade (p=.0007) initial metastatic status (p=.005) and vasculonervous or bone involvement (p=.04). For 145 pts M0 at entry, DFI was correlated with tumor grade (p=.0016), complete surgical excision (p=.0001) and radiotherapy (p=<.0001); MFI was correlated with tumor grade (p=.0074) and a non liposarcoma histology (p=.03); LRFI was correlated with adjuvant radiotherapy (p<0.0001), complete excision (p=0.0001) and tumor grade (p=0.005).

*Conclusion*: If surgery and a complete tumor removal remains the cornerstone of the treatment in retroSTS, radiotherapy may contribute to local control. Moreover, although local control remains essential, a high risk of metastatic disease was apparent.

### EXPRESSION OF THE MET/HEPATOCYTE GROWTH FACTOR RECEPTOR GENE IN BENIGN MUSCULOSKELETAL TUMORS

*Riccardo Ferracini, Marina Martano, Nicola Baldini, Katia Scotlandi, Jay Wunder, Eric Masterson*

*Institute for Cancer Research, St. Prov. 142, Km 3.95, 10060 Candiolo, Italy*

Overexpression of the hepatocyte growth factor receptor (*Met*/HGF receptor), a transmembrane tyrosine kinase encoded by the *Met* proto-oncogene, has been involved in transformation and invasive behaviour of human carcinomas and sarcomas. We have previously found that bone sarcomas express high levels of HGF receptor while in some cases the ligand was co-expressed with the receptor, activating an autocrine loop. In this study we analyzed 35 bioptic samples of benign bone tumors for the expression of the *Met* proto-oncogene. The lesions included osteoblastomas, chondroblastomas, non ossifying fibromas, giant cell tumors and desmoplastic fibromas of bone. The snap frozen samples were tested by immunohistochemistry and Western blotting with anti-*Met* antibodies or with RT-PCR for *Met* and HGF mRNA. About 50% of all cases scored positive for *Met* expression. Although non statistically relevant, we report a trend of *Met* positivity in samples from recurrent or locally aggressive lesions. Sporadic co-expression of the *Met* receptor and ligand (HGF) was demonstrated. The possible role of the *Met* receptor in the pathogenesis of benign bone neoplasms suggests an early involvement of the oncogene in sarcoma transformation. This is in contrast to the biology of carcinomas where the *Met* oncogene is amplified as a late event in the neoplastic progression.

### MANAGEMENT OF EXTRA-ABDOMINAL DESMOID TUMOR

***G. I. Castellarin, G. Barbanti-Bròdano, G. Pignatti, N. Baldini***

*Istituti Ortopedici Rizzoli, 40136 Bologna, Italy*

This study includes a continuous series of 103 patients with extra-abdominal desmoid tumor who were treated at the same institution between 1970 and 1996. Twenty patients were excluded from the analysis because of an insufficient follow-up. Of the remaining 83 patients, 35 had a newly diagnosed lesion, whereas 48 presented with a local recurrence after being surgically treated elsewhere. Patients were treated only with surgery (n=63) or with surgery plus radiation therapy (n=17) at doses ranging 3500 to 6600 cGy. Three patients did not undergo surgery, and were excluded from the analysis. No patient died of the disease, but 37 (46%) developed local recurrences. More than three recurrences in 8 patients (10%). Overall, relapses were not associated with age, sex, site, or tumor size, whereas a trend toward a higher risk of recurrence was observed when surgical margins were found to be inadequate on pathologic examination. The relapse rate was similar in patients treated only with surgery (28 cases, 45%) and patients treated with surgery plus radiation therapy (7 cases, 41%). Among the 35 patients who were first treated at our institution, 13 (37%) developed a recurrence. Again, no difference in the relapse rate was observed among patients undergoing surgery plus radiation therapy (2/5 cases, 40%) and patients treated only with surgery (11/30, 37%), whereas inadequate (intralesional or marginal) surgical margins were associated with a higher incidence of relapse (7/15, 47%) compared to patients treated with a wide surgical excision (7/20, 35%). These data confirm that the best chances to prevent the risk of local recurrence in extra-abdominal desmoid tumor are offered by a careful preoperative staging in order to obtain adequate surgical margins. Proper evaluation of the final specimen is mandatory to confirm the adequacy of surgery, although postoperative radiation therapy does not appear to be able to prevent the risk of local recurrence. Clinical observation should be considered for slowly growing recurrences.

### SUPPORT FOR LOCAL TREATMENT MODALITIES IN PATIENTS WITH AGGRESSIVE FIBROMATOSIS

***R. Keus, F. van Coevorden***

*The Netherlands Cancer Institute, Antoni van Leeuwenhoek Huis, Plesmanlaan 121, 1066 CX Amsterdam, The Netherlands*

*Background and Objectives*: Aggressive fibromatoses (desmoid tumors) are rare soft tissue neoplasms with a typical clinical behavior of frequent local recurrences and absence of distant spread. Other characteristics of this disease are an age peak between 30 and 40 years, female predominance often with pregnancy associated abdominal wall lesions. Mesenteric lesions are frequently seen in patients with an APC gene mutation as in familial polyposis coli. Local treatment modalities such as surgery and radiotherapy form the mainstay of the management of these tumors. In patients where local treatments are not feasible or failed, systemic therapy by hormonal manipulation or chemotherapy regimens is employed. The objective of this study was to determine the efficacy of local treatment modalities and to estimate the need for systemic treatment in our center.

*Methods*: A retrospective review of 105 patients (72 female, 33 male) in the period 1973 to 1997. Mean age was 33.1 years. Tumors were located in the head and neck in 11 patients, the extremeties in 50, abdominal wall in 24, mesenteric in 6 and other trunk in 12. Treatment consisted of surgery only in 21 patients, combined therapy with surgery and radiotherapy in 62, radiotherapy only in 6, isolated limb perfusion with Adriamycine and Melphalan in 4, chemotherapy in 3. Observation after surgery elsewhere was performed in 9 patients.

*Results*: Overall there was 86% local control at 5 years. There was no difference in local control for primary vs. recurrent lesions. Local control by treatment modality was 83%, 89%, 83% and 78% for surgery only, surgery and radiotherapy, radiotherapy only and other treatments, respectively. For the different tumor sites the were 100%, 81%, 88%, 67% and 100% in head and neck, extremities, abdominal wall, mesenteric and other trunk, respectively. No effect on local control of age, radiation field size, radiation dose was seen. The influence of surgical margin on local control rate in the surgery only group was 92% vs. 50% and in the combined therapy group 100% vs. 85% for patients with negative vs. positive surgical margins. Severe complications were seen in 14 patients: radiation myelitis 1, nerve injury 4, vascular injury 2, bone fracture 2, intestinal fistula 3, chronic infection 1, post-irradiation sarcoma 1. The outcome of chemotherapy in 3 primary and 3 recurrent lesions was in 1 CR, 2 PR, 1 NC and 2 PD. Tamoxifen was applied in 4 patients with recurrent lesions resulting in 1 CR, 1 NC and 2 PD.

*Conclusions*: These results show that local treatment with surgery and/or radiotherapy is effective in 86% of patients with aggressive fibromatosis. Only patients with extensive or multiple lesions not suitable for local treatment, or those who will be likely to suffer from unacceptable morbidity from local treatment should be considered for future studies of more effective systemic treatment.

### MDR1 GENE EXPRESSION AND OUTCOME IN OSTEOSARCOMA: A PROSPECTIVE INTERNATIONAL MULTICENTER STUDY

***J. S. Wunder, R. S. Bell, I. L. Andrulis, A. M. Davis, S. B. Bull, C. P. Beauchamp, E. U. Conrad, R. J. Grimer, J. H. Healey, M. G. Rock***

*University Musculoskeletal Oncology Unit and Samuel Lunenfeld Research Institute, Mount Sinai Hospital, University of Toronto, Toronto, Canada M5G 1X5*

Chemotherapy resistance remains an important clinical problem in osteosarcoma. This prospective, multicenter study analysed the prognostic value of multidrug resistance gene (MDR1) expression in patients with extremity, non-metastatic, conventional, high grade osteosarcoma. Between 1989 and 1994, 123 patients received adriamycin-based multiagent neoadjuvant chemotherapy and locally curative surgery, and were followed for a minimum of 24 months or until systemic recurrence. For each patient, a sample from the primary tumor was analysed for MDR1 expression level using a quantitative polymerase chain reaction-based assay.

MDR1 level was low in 44 tumors, intermediate in 39 tumors and high in 40. Of 123 patients, 46 (37%) relapsed with metastases at a minimum 2-year follow-up. After univariate analysis, only tumor size (p=0.0007) and chemotherapy-induced tumor necrosis (p=0.031) were associated with a higher risk of metastases. There was no linear association between MDR1 expression level and outcome (p=0.62). Surprisingly, patients with Intermediate MDR1 levels had the best survival, compared to those with either Low or High MDR1 expression. This relationship remained unchanged even after adjusting for other variables. After multivariate analysis, tumor size was the only significant predictor of outcome (p=0.004). As well, no relationship existed between MDR1 level and chemotherapy-induced tumor necrosis.

These results suggest that there is no simple linear relationship between MDR1 gene expression and systemic relapse in osteosarcoma. Patients whose tumors had High MDR1 expression did worse than those with Intermediate levels. On the other hand, Intermediate levels predicted for a better outcome than Low levels. If MDR1 expression is important in osteosarcoma, other mechanisms may play a more critical role. Studies using immunohistochemistry (IHC) have suggested that elevated P-glycoprotein levels are associated with a poor outcome. These results are in contrast to our data which show that patients whose tumors had low MDR1 levels had a worse prognosis. To help clarify this, we are presently evaluating a subset of tumors in our study of P-glycoprotein status by IHC. Mutation of the p53 gene might be another potential mechanism affecting outcome. Mutant p53 has been suggested to cause tumor drug resistance, and may also stimulate the MDR1 promoter, while wild-type p53 may act as a repressor. The interaction between these two genes may be important, and is also being investigated.

### COMBINED THERAPY FOR RETROPERITONEAL SARCOMA (RPS): RADIATION DOSE ESCALATION WITH POSTOPERATIVE BRACHYTHERAPY (BT)

***C. J. Swallow**, C. N. Catton, B. O'Sullivan, J. Couture, R. A. Kandel*

*The Sarcoma Site Group, University of Toronto, Canada*

*Introduction*: The anatomical constraints of the retroperitoneal space make adequate resection and effective irradiation of RPS particularly challenging, and treatment outcome is far worse than for sarcomas at other sites. We have recently implemented a new treatment protocol for RPS which involves aggressive en bloc total gross resection combined with escalated dose radiotherapy to the tumor bed using external beam radiotherapy (RT) and postoperative BT. Treatment outcome has been studied prospectively.

*Methods*: Over an 18 month period, 32 patients with primary or recurrent RPS were referred to our center. Of these, 18 were eligible for curative therapy with combined surgery and radiation. Sixteen patients completed all phases of therapy, while 2 who developed metastases during RT were taken off protocol. Sixteen patients had a total gross resection. Fourteen were treated with RT and BT, and 2 patients treated for recurrence after prior RT had BT only. RT was given preoperatively (n=12) or postoperatively (n=2) with a parallel pair to a dose of 45 or 50 Gy in 25 fractions over 5 weeks. Planar pulsed dose rate BT was administered postoperatively at 0.5 Gy/hr prescribed at 0.5 cm through temporary catheters implanted intraoperatively. The median total combined dose to the tumor bed was 70 (60–77) Gy. Outcome was assessed prospectively with toxicity scored according to the RTOG system.

*Results*: Of the 16 patients who completed all phases of therapy, 9 had a primary RPS and 7 presented following 1–3 recurrences. Median tumor diameter along the largest axis was 12 (4–28) cm. All patients had a total gross resection. Adjacent structures completely or partially resected included psoas/iliacus (63%), large bowel (63%), kidney/adrenal (63%), chest wall/diaphragm (31%), liver (31%), pancreas (19%), stomach (19%), spleen (19%), and small bowel (12%). The median area implanted was 68 cm2 (30–96 $cm^2$).

Median postoperative follow-up is 14 (4–24) months. The highest acute RTOG score after RT was 2, in 6 patients. After BT, 5 patients required parenteral nutritional support for transient duodenitis or enteritis and had an acute score of 3. One patient developed respiratory complications requiring early termination of BT and transfer to the ICU, and 1 patient died of complications following aspiration pneumonia. In 13 evaluable patients, the late toxicity score is 0 in 10, and 3 in 2. At a median of 14 months, one patient has failed with out-of-field mesenteric seeding, while 12 patients remain disease-free.

*Summary*: This study demonstrates the feasibility of combining an aggressive resection with high doses of radiation to the tumor bed for primary or recurrent RPS. Resection of involved adjacent structures produced a resection rate of 100%. Treatment-related mortality was 6%. Postoperative complications and acute radiation toxicity were manageable in the majority of cases. Preoperative RT was very well tolerated, and the late radiation toxicity from combined RT and BT was significant but acceptable. The efficacy of this approach will be tested in a phase II trial.

### A BIOLOGICALLY RELEVANT MODEL OF OSTEOSARCOMA WITH SPONTANEOUS PULMONARY METASTASES

***C. Khanna**, L. Helman*

*Pediatric Oncology Branch, National Cancer Institute, National Institutes of Health, Bethesda, MD 20892, USA*

Osteosarcoma (OSA) is the most common primary tumor of bone. Despite successful control of the primary tumor and adjuvant chemotherapy, relapse of OSA in the lungs occurs in 30% of patients within 5 years. A relevant animal model for OSA is needed to evaluate novel treatments and to identify key determinants for lung metastases. The objective of this work was to develop a model of OSA in an immunocompetant host, with primary tumor growth at orthotopic sites, spontaneous pulmonary metastases and variants differing in pulmonary metastatic potential. Hypothesis generation in these studies was based on the premise that tumor growth and metastasis are influenced by the host, the microenvironment and the tumor.

Murine OSA cell lines (K-7 and K-12), derived from a spontaneous murine OSA (Schmidt et al), were delivered at ectopic and orthotopic sites to 4 wk female balb/c mice. Sites for tumor cell line (or enzymatically digested tumor tissue) injection included subcutaneous, intramuscular (IM), intratibial, and intrafemoral. Sites for tumor fragment implantation included proximal tibial bone flaps or cranial tibial muscle flaps. Endpoints included primary tumor growth, bone invasion, radiographic change and incidence of lung metastases. The influence of tumor-bearing-limb amputation on lung metastases was studied. Lung metastases from K-7 tumors were surgically re-implanted to tibial bone flaps. Repeating this lung to bone passage two times, yielded the K-7M2 variant. Features of K-7M2 were evaluated as above.

From the experimental variables studied the optimal model system consisted of surgical implantation of tumor tissue at a tibial muscle flap or IM injection of enzymatically digested tumor followed by amputation of limbs bearing 1 cm tumors. using this model system, K-7 tumors developed in 12 days in 93% of mice, growing to 1 cm in 19 days. K-12 tumors developed in 12 days in 87% of mice, growing to 1 cm in 26 days. After amputation pulmonary metastases occurred in 100% of K-7 mice compared to 58% of K-12 mice. The median survival for K-7 mice was shorter than K-12 (61 days compared to 138 days; p<0.01. K-7M2 had primary tumor growth similar to K-7, but earlier death from lung metastases (median survival 48 days).

This biologically relevant OSA model will offer a valuable tool for the evaluation of novel OSA treatment strategies and will improve our understanding of OSA metastases. This model is characterized by orthotopic primary tumor growth in an immunocompetant animal, spontaneous lung metastases, and variants with a pulmonary metastatic gradient from most aggressive to least aggressive (K-7M2, K-7, K-12). The osteosarcoma model developed will offer a valuable tool for the evaluation of novel treatment strategies for osteosarcoma and for the examination of determinants of osteosarcoma metastases. Studies are underway to identify genetic determinants that may account for differences in the metastatic potential of the K-7, K-12, and K-7M2 models. Results of this model characterization and the ongoing genetic studies will be presented.

### GROWTH INHIBITION OF LEIOMYOSARCOMA *IN VIVO* USING AN ADENOVIRUS OVEREXPRESSING THE TRANSCRIPTION FACTOR E2F-1

*N. Hetrakul, A. Abramanian, T.-J. Liu, M. Wilson-Heiner, W. Xia, N. Mirza, D. Yu, R. Pollock, B. Feig, P. Pisters, K. Hunt*

*The University of Texas M.D. Anderson Cancer Center, Houston, Texas 77030, USA*

We have previously shown that overexpression of the transcription factor E2F-1 results in apoptotic cell death in leiomyosarcoma cells lines *in vitro*. This cell death appears to be independent of the *p53* status of the cells. We hypothesized that overexpression of E2F-1 in established tumors would result in growth inhibition *in vivo*.

*Methods*: The leiomyosarcoma cell line SKLMS-1 was injected into the flank of Balb/c nu/nu mice at $5 \times 10^6$ cells per animal. Once tumors reached 5 mm size, tumors were injected with PBS (6 mice), a recombinant adenovirus expressing the Luciferase reporter gene (Ad5Luc; 6 mice) or a recombinant adenovirus expressing E2F-1 (Ad5E2F; 7 mice). Ad5Luc and Ad5E2F were injected at a dose of $2 \times 10^9$ pfu every other day for a total of 13 injections. Tumor growth was measured in 3 dimensions and recorded as tumor volume ($mm^3$). Mice were sacrificed when tumors reached 20 mm in size and assessed for apoptosis using *in situ* TUNEL assay. E2F expression was evaluated using immunohistochemistry.

*Results*: We noted marked reduction in tumor growth in the Ad5E2F treated tumors over controls (PBS and Ad5Luc). Please see graph below.

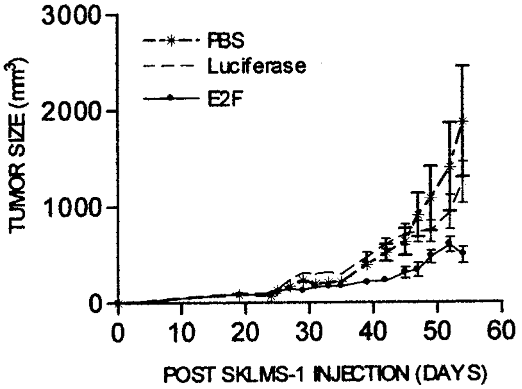

Tumor growth in the Ad5E2F treated animals was significantly reduced as compared to Ad5Luc treated or PBS treated controls (P=<0.0001) by ANOVA. There was complete tumor regression in two animals. In situ TUNEL demonstrated apoptosis in Ad5E2F treated tumors in 20–30% of cells per high power field examined. Overexpresssion of E2F was confirmed using immunohistochemistry and was predominantly nuclear.

*Conclusions*: Adenovirus mediated overexpression of E2F-1 leads to nuclear localization of E2F-1 with resultant apoptotic cell death in leiomyosarcoma. *In vivo* gene therapy utilizing E2F-1 results in significant growth inhibition and may prove to be a useful strategy in the clinical management of leiomyosarcoma.

### PREOPERATIVE TAILORED RADIATION THERAPY FOR ADVANCED RETROPERITONEAL SOFT TISSUE SARCOMAS. A FEASIBILITY STUDY

*Alessandro Gronchi, Alberto Azzarelli, Marcello Zanini, Lorenza Gandola, Susanna Fissi, Paolo Casali, Rossella Bertulli*

*Istituto Nazionale Tumori, Via Venezian 1, 20133 Milano, Italy*

*Purpose*: Retroperitoneal soft tissue sarcomas (RSTS) are generally large lesions, often recurrent and involving viscera, which are difficult to be treated properly with surgery. For postoperative radiation therapy (RT) to be effective, one should employ doses and fields which are unfeasible. In an effort to improve results, we decided to assess the feasibility of an approach including preoperative RT. In this way, RT fields can be tailored to the lesion, and the lesion itself works as a spacer able to protect viscera, while surgery could take advantage from a response to RT.

*Materials and methods*: Since September '96 to December '97, 12 consecutive patients with extensive recurrent RSTS were treated with preoperative RT up to 50 Gy. Surgery was scheduled between the 30th and 60th day after the end of RT. The fields of RT were designed upon an isodose simulation CT scan, especially targeted to the portions of the lesion for which good margins would have been difficult to be achieved, also considering whether colectomy or nefrectomy would have been performed or not. The end points were the overall feasibility of the treatment program, the intraop. and postop. complications, the pathologic response rate and the local control.

*Results*: Twelve pts. were treated: 8 liposa. (3 G1), 3 leiomiosa. (2 G1), 1 NOS sarcoma. One pt. received only 32 Gy for gastrointestinal intolerance. Radiological response was minimal in all pts. All the patients underwent surgery 32–46 days later. In ten patients, the lesion was removed completely. Resections were extended to viscera, with 4 nefrectomies and 6 colectomies. No major surgical difficulties were encountered, and the lesions always appeared well demarcated. No surgical contamination did occur. Postoperatively, 4 of 6 colectomies were complicated by anastomotic fistulas, and two required colostomy. One pt. died three months later due to abdominal complications. One pt. died due to disease recurrence. One pt. was reoperated for disease recurrence 10 mos later. Pathologic response was always consistent with post-RT alterations. Up to now (follow-up range 8–20 mos), all the other seven patients are alive with no evidence of disease.

*Conclusion*: Preoperative RT is feasible in RSTS. In the case a large bowel anastomosis is required, precautional colostomy or ileostomy should be considered.

### HIGH-DOSE IFOSFAMIDE AND DOXORUBICIN (HDI-DX) IN ADVANCED PREVIOUSLY UNTREATED SOFT TISSUE SARCOMA (STS) PATIENTS. A PHASE II STUDY OF THE SPANISH GROUP FOR RESEARCH ON SARCOMAS (GEIS)

***A. López-Pousa**, J. Montalar, J. M. Buesa, J. Maurel, J. Martin, J. Cassinello, I. Sevilla, R. De las Peñas, J. Cruz, C. Balana, J. García del Muro, A. Povedo*

*Spanish Group for Research on Sarcomas, Hospital de Sant Pau, Avda Padre Claret 167, 08025 Barcelona, Spain*

*Introduction*: Doxorubicin (DX) and high-dose Ifosfamide (HDI) are two active drugs in the treatment of STS with a similar response rate. We are performing a phase II trial in first line treatment with HDI-DX (scalating dose of HDI), in order to evaluate the activity and toxicity of this regime.

*Material and methods*: Since Jul 97 we have included 54 previously untreated advanced STS pts in a trial with HDI-DX, with a fixed dose of DX 50 mg/m$^2$ and a continuous infusion of HDI, starting at 12 g/m$^2$ and scalating dose to 13 and 14 g/m$^2$ (2 g/m$^2$ in 2-hours infusion d1, followed by 2 g/m$^2$/day d1 to d5–6), in absence of any grade IV toxicity or neutropenic fever, per cycle in each patient. Otherwise a dose reduction to 10 g/m$^2$ has been allowed. GM-CSF (Leucomax (Schering-Plough) 5 μg/Kg/day × 7 days has been administered after the end of each cycle of chemotherapy.

Thirty-five pts are already evaluable: 16 male, 19 female; median age 55 (27–65) years. Performance status: WHO grade 0–13; 1–14; 2–8 pts. Histology: leiomiosarcoma 9, malignant fibrous histiocitoma 5, synovial 3, liposarcoma 3, fibrosarcoma 3, neurogenic 3, mixed müllerian 2, others 7. Target tumor location: non-resectable primary or recurrence 18 (exclusively 10), lung 16, nodal 2, liver 2, soft tissue 1, peritoneal 1 and bone 1. Histologic grade: 1–6; 2–9; 3–17 pts.

*Results*: We have already administered 87 evaluable cycles (cy), median 2 (range 1–5). Dose intensity of HDI has been 2.80 (1.7–3.5) g/m$^2$/week. Dose of HDI per cycle has been: 12 g/m$^2$ in 51 cy, 13 g/m$^2$ in 12 cy, 14 g/m$^2$ in 5 cy and 10 g/m$^2$ (reduction dose) in 19 cy.

Haematologic toxicity (grade 3–4 CALBG—% cy): Hbne 17%, Leuc 13% G3, 37% G4; Gran 9% G3, 41% G4; Plat 14% G3, 12% G4.

Non-Haematologic toxicity (grade 3–4 CALBG—% cy): Nausea 13%; Vom 14%; Stomatitis 9% G3; Hematuria 1% Neurocortical 5% G3, moderate somnolence/blurred vision 9%; Infection 18% G3, 5% G4; Asthenia 19% G3, 2% G4; Anorexia 15%; Cardiac 3.5% G3. Thirteen pts have presented neutropenic fever in 20 cy (23%). There was one toxic death due to a septic shock after the first cycle of chemotherapy. RBC transfusions were required in 22% cy and plt transfusion in 5% cy. Objective activity: 8 PR, 7 SD, 5 PD, 8 NE in 28 evaluable pts.

*Conclusions*: HDI-DX is an active regime in adult STS. The impact of haematological toxicity may limit the use of this schedule and it is too early to evaluate the cardiac and neurological toxicity.

### HIGH DOSE CHEMOTHERAPY FOR SOFT TISSUE EWING'S SARCOMAS

*R. E. Hough, S. Browne, L. M. El-Helw, R. E. Coleman, **M. H. Robinson**, P. C. Lorigan*

*Yorkshire Cancer Research Department of Clinical Oncology, Weston Park Hospital, Sheffield S10 2JS, UK*

Soft tissue Ewing's sarcoma, peripheral neuroectodermal tumours (PNET) and Askin's tumours are small round blue cell tumours which characteristically have a reciprocal translocation between chromosome 11 and 22. As with other rare tumours, clinical trial data on the optimal management of these patients is limited. We review the results of 6 patients treated with conventional chemotherapy and compared these to 9 patients treated with standard induction regimens followed by high dose chemotherapy (HDC).

6 patients (1 female, 5 male) received standard chemotherapy with IVAD (or similar regimen). Mean age at presentation was 23 years (range 16–33 years). 2 had localised disease, 2 locally advanced and 2 metastatic disease. Additional radical radiotherapy was given to 5 patients and surgery was performed in the other. 4 patients have died at a median time of 284 days. Survival in the 2 remaining patients is 1947 and 2006 days.

9 patients received IVAD (7 patients) or EVAIA (2 patients) induction chemotherapy. Mean age at presentation was 26 years (range 17–39 years). 3 had locally advanced disease with the remainder having metastatic disease. Complete response (CR) was achieved in 2 patients and a good partial response (PR) was seen in all others. HDC in all patients was with Etoposide, Carboplatin and Melphalan and a further 3 CR were achieved. Additional radical radiotherapy was given to 6 patients. 6 patients have died after a median interval of 421 days. 3 patients are alive and in remission at 417, 666 and 884 days.

Soft tissue Ewing's sarcoma often responds to conventional chemotherapy but tends to relapse, and the long term prognosis is poor. HDC may offer some survival advantage but multi-centre clinical trials of this and newer strategies will be vital in improving the outlook for these patients.

### SUCCESSFUL PHASE II TRIAL OF ETOPOSIDE AND HIGH DOSE IFOSFAMIDE IN NEWLY DIAGNOSED METASTATIC OSTEOSARCOMA: PRELIMINARY REPORT. A PEDIATRIC ONCOLOGY GROUP TRIAL (POG)

*A. M. Goorin, P. Gieser, W. Ferguson, M. Harris, M. Link, M. Gebhardt, G. P. Siegal, R. C. Shamberger, M. Bernstein, C. Schwartz, H. Berrey, H. E. Grier*

*Pediatric Oncology Group, Chicago, IL, USA*

Patients with metastatic osteosarcoma (MOS) at presentation have a long term survival of <30% (POG 9259). The use of the most active agents in MOS have all produced tumor regression in only 20–30% of patients with MOS. More effective chemotherapy to treat MOS is needed. An MTD of 500 mg/M$^2$ of etoposide (VP), and 17.5 gms/M$^2$ of ifosfamide (IFOS) was determined in POG 9170 for relapsed MOS (cumulative cisplatin dose ≤300 mg/M$^2$). That trial had a response rate (RR) of 6 of 13. Thus a phase II trial was initiated. The objective was to estimate the RR and define toxicity of VP, IFOS, and G-CSF in newly diagnosed MOS. The study was closed September 15, 1997 having reached its accrual goal. A total of 43 patients were registered. Two patients were ineligible; 2 were not evaluable; and 39 were evaluable for response. Patients received infusions of 100 mg/M$^2$/day of VP over 1 hour followed by 3.5 gr/M$^2$/day of IFOS over 4 hours for 5 days total. G-CSF, was began on day 6. This was repeated in 3 weeks. Response was determined at week 6. Eligibility included: age ≤30 years, biopsy proven newly diagnosed, previously untreated MOS, and ECOG performance status ≤2. Twenty-eight of 41 (68%) have metastatic sites only in the lung and 13 (32%) have synchronous MOS with metastatic sites in other bones. Toxicity was evaluated in 38. The most serious toxicities are; grade 5 sepsis (death) in 1; grade 3 and 4 sepsis in 6; grade 3 and 4 infections (non-sepsis) in an additional 5. One patient died of congestive heart failure (CHF), and a second patient had grade 1 CHF. Grade 4 neutropenia occurred in 30 and Grade 4 thrombocytopenia in 18. Grade 3 Fanconi's Syndrome developed in 4 and one additional patient had grade 4 Fanconi's. Response information is available on 38 patients. Three had CR's; 20 PR's; 3 MR's; 10 NR; 2 PD. The RR (CR + PR) is 61% ± 8% (SE). We conclude that the combination of VP and high dose IFOS are very effective induction treatment for high risk MOS despite significant associated myelosuppression sometimes complicated by infection, and renal toxicity requiring electrolyte replacement.

### PEGYLATED LIPOSOMAL DOXORUBICIN (DOXIL™) IN REFRACTORY SARCOMA

*K. M. Skubitz*

*University of Minnesota Medical School, Minneapolis, MN 55455, USA*

There is interest in administering chemotherapy drugs in liposomes as a means of altering the therapeutic index of the drug. Liposomal doxorubicin is of particular interest since less cardiotoxicity has been observed, and there may be greater anti-tumor efficacy. Pegylated-liposomal doxorubicin (Doxil™) is a unique form of liposomal doxorubicin in which the liposomes are coated with methoxypoly (ethylene glycol). The polymer confers useful properties including a diminished uptake by the reticuloendothelial system, leading to a much longer half-life in blood (~50–60 hours), and a different toxicity profile than non-pegylated liposomes. We are performing a phase II study of Doxil™ in refractory sarcoma. The patient population consists of patients that have failed doxorubicin, DTIC, ifosfamide, VP-16, and in some cases additional chemotherapy. The initial dose per course is 55 mg/m$^2$ every four weeks. Dose modification based on mucositis and hand-foot syndrome (the main limiting toxicities) is performed following an algorithm that modifies both dose and interval between treatments. Treatment was generally well tolerated. Of the first 29 evaluable patients, there were: 5 osteosarcomas, 7 gastrointestinal leiomyosarcomas, and 17 other soft tissue sarcomas. Eight had stable disease for 14, 7, 6, 3, 3, 3+, 2+, and 2+, months; 1 a mixed response, 3 a minor response for 3+, 4, and 9 months, and 2 a partial response (maintained for 5, and 24+ months). These data suggest that pegylated-liposomal doxorubicin has significant activity in refractory sarcoma.

### REPAIR OF RADIATION-INDUCED DNA DOUBLE STRAND BREAKS IN HUMAN FIBROBLASTS

*J. Aronowitz, B. Nevaldine, P. Hahn*

*State University of New York Health Science Center, Syracuse, NY 13204, USA*

*Introduction*: Little is known regarding the repair kinetics of radiation-induced DNA double strand breaks (DNAdsb), especially in normal tissue. With increasing interest in assaying normal tissue radiosensitivity, as well as in optimizing time-dose-fractionation schemes to maximize therapeutic ratio, we endeavored to develop techniques to quantify normal fibroblast repair kinetics.

*Materials and methods*: Non-transformed cultured fibroblasts from a normal human subject were studied. It was determined that terminal differentiation occurred after 18 passages. Utilizing a system based on pulsed-field gel electrophoresis, we measured repair kinetics of cohorts of cells from the 7th and 15th passages following irradiation with 7.5 Gy.

*Results*: Approximately 50% of DNAdsb were repaired within minutes (fast repair), an additional 30% before the end of the first hour, and most of the remaining breaks were repaired during the second and third hours. The $T^{1/2}$ of slow repair during the first hour was 40 minutes. There were no appreciable differences in the repair kinetics between fibroblasts from early (7th) and late (15th) passages.

*Conclusion*: The repair kinetics of DNAdsb in human fibroblasts can be determined. In this culture of normal human fibroblasts, repair kinetics were not altered by the age in culture.

### PHASE II TRIAL OF ONCONASE® (ONC) IN PATIENTS WITH ADVANCED MALIGNANT MESOTHELIOMA (MM): ANALYSIS OF SURVIVAL

*R. N. Taub, M. L. Keohan, R. L. Fine, J. Constanzi, Z. Darzynkiewicz, H. Chun, A. Mittelman, T. Panella, S. McCachren, C. Puccio, K. Shogen, S. M. Mikulski*

*Columbia University, New York, NY [RNT, MLK, RLF], Lone Star Oncology, Austin, TX [JC], New York Medical College, Valhalla, NY [ZD, HC, AM, CP], Thompson Cancer Survival Center, Knoxville, TN [TP, SM], Alfacell Corporation, Bloomfield, NJ [KS, SMM], USA*

Systemic therapy for advanced, unresectable MM remains unsatisfactory. No single or combination drug regimen has been shown to prolong survival. We have conducted a clinical phase II trial of ONC (previously known as P-30 protein), a novel ribonuclease isolated from the eggs of the leopard frog (*Rana pipiens*), in patients with advanced unresectable MM. ONC had previously demonstrated *in vitro* activity against human primary mesothelioma cell cultures. In this multicenter open label trial, single-agent ONC was given at a dose of 480 $\mu g/m^2$ I.V. weekly (as a 30-min infusion) to 105 enrolled patients (85M:20F, performance status 0–2) until disease progression or intolerable toxicity. 92 pts had prior surgery (none had complete resection), 17 prior radiotherapy, 39 prior chemotherapy. Involvement of the pleura/lung was noted in 97 pts, including 17 with pleural+peritoneal disease; 8 pts had peritoneal disease only. 38 pts had regional lymph node involvement, and 33 had distant metastasis. Toxicities were reversible and manageable. 16 pts were terminated from study due to toxicity, 4 of which for grade 4 toxicities. As of 6/15/98, the estimated Kaplan-Meier median survival times (MSTs) for the intent-to-treat group (105 pts) was 5.8 months (1 and 2 year survival, 34.3% and 20.0%). Patients with confirmed epithelioid histology (50/105) fared better (MST 9.6 months, 1 and 2 year survival, 42% and 23.4%) than pts with pure sarcomatoid histology (8/105 pts, MST 1.9 months, 1 year survival 12.5%). Age under 50 (17/105 pts, MST 19.8 months), female sex (20/105 pts, MST 11.4 months) and performance status of 0 (29/105 pts, MST 18.4 months) were favorable criteria for survival. Our 1 and 2 year survival data compared favorably with those of analagous prognostic groups as defined by the Cancer and Leukemia Group B (CALGB) among 337 pts treated by CALGB with different Phase II agents or regimens over a 10-year period. Our data indicate that ONC may be active in selected patients with MM. A randomized Phase III trial of ONC v. doxorubicin is now in progress.

### BLOCKAGE OF INSULIN-LIKE GROWTH FACTOR I-RECEPTOR IN EWING'S SARCOMA: A PROMISING BASIS FOR INNOVATIVE THERAPY

***K. Scotlandi*, S. Benini, M. Serra, M. C. Manara, P. Picci, N. Baldini**

*Laboratory of Oncology Research, Istituti Ortopedici Rizzoli, 40136 Bologna, Italy*

Innovative treatment modalities are needed for Ewing's sarcoma (ES), a neoplasm with a disappointingly low survival rate despite the use of aggressive multimodal therapeutic approaches. We have previously shown the existence and the pathogenetic relevance of an autocrine loop, mediated by the insulin-like growth factor-I receptor (IGF-IR), that is crucial for survival and proliferation of ES cells *in vitro*. In this study, we report that the IGF-IR-blocking monoclonal antibody αIR3 may also significantly inhibit ES cell growth *in vivo*. In particular, in almost one-half of animals tested, blockage of IGF-IR by αIR3 induced a complete regression of tumors developing after s.c. inoculation of ES cells, suggesting the value of IGF-IR as a specific target for novel therapeutic strategies. Also suramin, a drug that is able to interfere with growth factor binding to their receptors, inhibited both the tumorigenic and the metastatic ability of ES cells, therefore offering as a promising agent to be combined with conventional cytotoxic drugs for the design of more effective therapeutic regimens. Moreover, both αIR3 and suramin treatment increased the antitumor *in vitro* effects of adriamycin and vincristine, two conventional chemotherapeutic drugs with a leader action on ES, producing additive growth suppression of ES cell lines. This result was obtained both by simultaneous and sequential treatments. Analysis of the proliferation rate and of apoptosis revealed that αIR3 antibody did not substantially affect the blockage of cell cycle in G2/M phase induced by adriamycin, whereas it significantly enhanced the induction of apoptosis. A synergistic and dose-dependent effect was indeed observed with regard to the percentage of apoptotic nuclei after treatment with αIR3 and adriamycin, indicating that the specific blockage of IGF-IR deprives ES cells of an important tools preventing apoptosis induced by chemotherapeutic agents. In conclusion, we showed that the blockage of IGF-IR by neutralizing antibody or by suramin may significantly inhibit the growth of ES cells both *in vitro* and *in vivo*, and that this treatment strategy greatly potentiates the antitumor activity of conventional chemotherapeutic drugs.

### METASTASECTOMY AND CHEMOTHERAPY FOR LUNG METASTASES FROM SOFT TISSUE SARCOMA. A RANDOMIZED PHASE III STUDY

***K. S. Hall*[1], A. N. Van Geel[2], R. Blum[3], T. Alvegård[1]**

*[1]Scandinavian sarcoma group (SSG), [2]European organization for research and treatment of cancer (EORTC), [3]Eastern cooperative oncology group (ECOG), Oslo, Norway*

Soft-tissue sarcoma tumours account for approximately 1% of all malignancies. Although most patients present with apparently localised disease about 50% will die from subsequent metastases. In 70% of all cases metastatic disease involves the lungs only. This observation has led to the concept of surgical treatment for lung metastases. By proper selection a 5-year survival of about 40% can be obtained by metastasectomyalone. The present study aims to assess if preoperative chemotherapy with Doxorubicin and Ifosfamide improves survival of patients scheduled for metastasectomy for pulmonary metastases.

The study was initiated in April 1996 as an intergroup study between SSG, EORTC and ECOG. Patients undergoing radical metastasectomy of 5 or fewer lung metastases will be randomized to receive pre-metastasectomy chemotherapy (treatment #1) or no chemotherapy (treatment #2). Patients with treatment #1 will receive 3 chemotherapy cycles before and 2 cycles after metastasectomy provided if there is either a complete or partial response on CT scan or if there is a histological response grade III or IV. For grade I tumours (no necrosis) the patient does not continue to post-operative chemotherapy. Each participating institution has to select one of the two proposed chemotherapy regimen (standard: Doxorubicin 50 $mg/m^2$+Ifosfamide 5 $g/m^2$ or intensified: Doxorubicin 75 $mg/m^2$+Ifosfamide 5 $g/m^2$+GCSF:granulocyte-macrophage colony-stimulating factor).

The principal end-point is overall survival. Secondary end-points are disease free survival and side effects. To detect an improvement of 15% in the 3-year overall survival, 340 patients need to be included in the study. Final analysis will be performed after observation of 190 deaths.

*Study status*: So far 5 institutions from 2 cooperative groups have entered 12 patients. A total of 12 EORTC investigators have obtained approval from their local ethical committee, and 3 additional centers have submitted the protocol for approval. From SSG a total of 16 centers have confirmed their interest in participation of the study. The ECOG has recently joined this intergroup protocol, and the SWOG is considering its participation.

*Conclusions*: The major problem of this study has been a slow inclusion rate. As concluded in an intergroup meeting last April 1998 in Leuven arranged by EORTC Soft Tissue and Bone Sarcoma Group it is important that thoracic surgeons are informed of this protocol since they often see these patients primarily. Currently work is ongoing to recruit the participation of additional centers (SWOG and MSK).

### p53 INHIBITS REPAIR OF DNA DAMAGE FROM γ-IRRADIATION IN HUMAN SARCOMA CELLS

***M. Milas, F. Ali-Osman, O. Akande, D. Yu, R. E. Pollock***

*The University of Texas M.D. Anderson Cancer Center, Houston, Texas 77030, USA*

*Introduction*: Mutations of the p53 tumor suppressor gene are the most frequent genetic abnormality in soft tissue sarcomas, a group of tumors frequently resistant to radiation. We have previously shown that wild type (wt) p53 status enhances radiosensitivity of human leiomyosarcoma cells. Consequently, we investigated the mechanisms by which wt p53 mediates this improved radioresponse.

*Methods*: All experiments were conducted using a human leiomyosarcoma cell line SKLMS, with a missense p53 mutation at codon 245, and derived stable transfectants expressing constitutive wt p53 (SKp53-2, SKp53-3), a temperature-sensitive p53 mutant (SKAla-1), or a neomycin-resistant marker gene (SKneo). After 0, 5, or 10 Gy irradiation, distribution of cell cycle phases was determined by flow cytometry, and apoptosis rate by TUNEL assay. DNA damage and repair following irradiation at these doses was quantified by a PCR-based assay which displays decreased amplification product when there is a damaged DNA template that cannot bind or extend PCR primers. In this case, the DNA template was an actively transcribed gene, glutathione-S-transferase π (GST-π), whose gene product catalyzes glutathione conjugation to various electrophilic compounds including both carcinogens and anticancer agents. Damage to the GST-π gene presumably reflects the potential damage to other actively transcribed genes in this cell line. Cells were assayed at various intervals between 0 min to 24 hrs after irradiation.

*Results*: There was minimal G1 and G2/M cell cycle arrest after irradiation in these cell lines. However, both the wt p53 containing radiosensitive cells (SKp53-2, SKp53-3) and mutant p53 radioresistant cells (SKLMS, SKneo) showed similar distribution of cell cycle phases after radiation. None of the cell lines had significant apoptosis levels before or after treatment. The amount of initial DNA damage was comparable between wt and mutant p53 cell lines at both radiation doses. In contrast, wt p53 expressing sarcoma cells were markedly less able to repair radiation-induced damage compared to their mutant counterparts after both 5 and 10 Gy irradiation. 24 hr after 10 Gy irradiation, only 11–15% of GST-π gene remained intact in wt p53 cells, compared to 50–100% in mutant p53 cells ($p<0.05$, t-test).

*Conclusion:* These results indicate that p53 may have an additional important function to inhibit DNA repair in injured cells where downstream p53 pathways of cell cycle arrest and apoptosis are not as prominent after cellular stress, thereby eliminating potential genomic damage from the population and enhancing the cytotoxic effect of radiation. These data support further investigation of p53 restorative strategies as a means to improve the therapeutic benefit from radiation in human sarcomas.

### BONE AND SOFT TISSUE PATHOLOGY CONSULTATION PRACTICE: A REVIEW OF 50 CASES

***B. M. Wehrli, V. L. Fornasier***

*Vancouver General Hospital, Vancouver, BC and Wellesley Central Hospital, Toronto, Ontario, Canada*

*Background*: Since 1995, all new oncology patients at the Princess Margaret Hospital (PMH), prior to their receiving treatment, are required to have their pathology reviewed by a designated PMH consultant pathologist. This review is performed both to confirm the existing diagnosis and to ensure consistency in grading and staging of tumors. Through an examination of this review process, we hope to gauge the demographic scope of our consult service, to ascertain the degree of efficiency and effectiveness of our existing consultation protocol and to determine specific areas which could be improved.

*Design*: A retrospective review of 50 cases, received by the soft tissue and bone review pathologist between February 27, 1998 and July 30, 1998, was performed. Each case consisted of the consultant's report, and either the original pathology report or a letter of request for consultation, as well as any glass slides available for review. Criteria examined included: hospitals originating consults, turnaround time, availability of original pathology report, the nature of material sent for consult purposes, the nature of the gross and microscopic descriptions of specimens from the originating hospitals, and the concordance of initial diagnosis with the consultant diagnosis.

*Results*: The 50 consultation cases originated from 28 different hospitals, with all cases originating within Ontario with the exception of 6 cases, 2 of which originated in Quebec, and one case each in Newfoundland, New Brunswick, Saskatchewan, and Alberta. Turnaround time ranged from one calendar day to 104 calendar days, with an average of 10 days. Of the 50 consultation cases, 40 (80%) were accompanied by copies of the original pathology reports and 10 (20%) were not. The specimens described in the dictation reports consisted of 15 resections, 20 excisional/incisional biopsies, 4 core biopsies, and one fine needle aspiration biopsy. Most gross and microscopic descriptions of the specimens received from the originating hospitals were adequate. Fifteen (41%) of 37 cases demonstrated diagnostic concordance, whereas 22 (59%) of 37 cases did not demonstrate such concordance. Of these 22 cases in which a diagnostic discrepancy was evident, 7 (32%) represented major non-concordance. In 5 of these 7 major non-concordant diagnoses, the referring diagnosis was malignant and the consultant diagnosis was benign, and in the remaining 2 cases, the referring diagnosis was benign and the consultant diagnosis was malignant. The remaining 15 (68%) of 22 non-concordant diagnoses were minor in nature, with all demonstrating concordance concerning the benign or malignant nature of the specimen.

*Conclusion*: The consultant's bone and soft tissue consultation practice receives varying specimens from a widespread area. The non-concordant diagnostic rate between referring pathologists and the consultant is considerable. Patients who are diagnosed with sarcomata, should have their pathology reviewed by a bone and soft tissue consultant prior to radical therapy.

### CT-GUIDED BRACHYTHERAPY—A REVIEW OF 22 PATIENTS TREATED WITH INTERSTITIAL IMPLANTS FOR SKELETAL TUMORS

*K. M. Lee, H. D. Suit, I. J. Spiro*

*Purpose*: CT-guided technology can be used to enhance precision of dose delivery in brachytherapy (BRT). This paper is a review of 26 CT-guided BRT performed on 22 patients with tumors at skeletal sites.

*Methods and materials*: The records of the 22 patients were retrospectively reviewed with regards to patient characteristics, histological diagnosis, treatment, BRT technique, tumor control, palliative benefit and complications. BRT was performed either intraoperatively or percutaneously with the use of CT imaging for placement of catheters.

*Results*: Between 1993 and 1998, 22 patients were treated with CT-guided BRT to 26 anatomical sites, mainly involving the axial skeleton. Histological diagnoses included chordoma, osteosarcoma, chondrosarcoma, adenocarcinoma and other connective tissue tumors. Nine patients presented with primary tumor while 13 had recurrent tumors including 11 with metastatic disease. Seven patients had previous irradiation at the BRT sites. Of the 15 BRT procedures performed with curative intent, there were 3 local failures including 1 with distant metastasis, 2 regional recurrence and 3 distant relapses. Of the 11 BRT procedures performed with palliative intent, there were 2 local failures also with distant metastasis, 1 regional recurrence and 4 distant relapses. The main palliative benefit was definite pain relief after 17 (71%) BRT procedures. The main BRT-related complications were wound problems seen after 5 (19%) BRT procedures.

*Conclusion*: CT-guided BRT can be used in the management of tumors at skeletal sites, in particular the axial skeleton where a high tumor dose can be delivered with a marked dose gradient through the spinal cord. This relatively new technique remains under further evaluation and undergoes constant fine-tuning.

### THE INTERGROUP RHABDOMYOSARCOMA STUDY GROUP (IRSG): PROGRESS AND PROPOSALS FOR THE NEXT STUDY, IRS-V

*R. B. Raney, J. R. Anderson, W. Crist, H. M. Maurer*

*IRSG of the Children's Cancer Group and Pediatric Oncology Group, UT MD Anderson Cancer Center, Houston, TX 77030, USA*

In 1972, members of the pediatric oncology cooperative groups formed the IRSG with support from the National Cancer Institute to study rhabdomyosarcoma (RMS) and undifferentiated sarcoma (UDS) in previously untreated patients less than 21 years of age. Since then, 3 successive protocols have shown improved overall 5-year survival rates, from 55%, in IRS-I to 71% in IRS-III (P<0.001; *J Clin Oncol* 1995;13:610). IRS-IV (1991–1997) tested a randomized comparison of vincristine, actinomycin D, and cyclophosphamide (VAC, with C=2.2 gm/m2/course) *vs.* VAI (ifosfamide, 9 gm/m2/course) *vs.* VIE (etoposide, 500 mg/m2/course) for patients with localized disease. Conventional radiotherapy (XRT; 50.4 Gray) was compared to hyperfractionated XRT (59.4 Gray) for patients with localized, gross residual sarcoma. Preliminary results should be available in 11/98. Proposals for IRS-V therapy are based on stratifying patients into 4 categories according to the probability of failure: low-risk, intermediate-risk with embryonal (EMB) RMS, intermediate-risk with alveolar (ALV) RMS or UDS, and high-risk patients with metastases (Stage 4) at diagnosis. Tumor specimens will be subjected to cytogenetic and molecular analyses, in order to ascertain whether prognostic subgroups can be identified among the patients with EMB and ALV RMS and UDS. The general strategy is to design therapy based on the likelihood of cure. The Table shows the four categories of patients, the 3-year failure-free survival (FFS) and overall survival estimates, and the types of treatment to be administered.

| Category | % 3-year FFS | % 3-year Survival | Treatment |
|---|---|---|---|
| Low-risk | 88 | 95 | VA ± CPM ± XRT |
| Intermediate EMB, including Stage 4 <10 yr. | 45–76 | 51–84 | VAC with ↑CPM + XRT |
| Intermediate ALV/UDS | 51–72 | 61–81 | VAC + Topotecan + XRT |
| High-risk EMB >10 yr. + ALV/UDS | 22 | 38 | New-agent window, then VAC + XRT |

Supported in part by Grants CA-24507 and CA-30138 from the National Cancer Institute.

### DESMOID TUMOR: OUTCOME AND PROGNOSTIC FACTORS FOLLOWING SURGERY, RADIATION, OR COMBINED SURGERY AND RADIATION

*M. T. Ballo, G. K. Zagars, A. Pollack, P. W. T. Pisters, R. A. Pollack*

*The University of Texas M.D. Anderson Cancer Center, Houston, Texas 77030, USA*

*Purpose*: To evaluate the therapeutic value of resection alone, radiation alone or combined modality therapy for desmoid tumors.

*Patients and methods*: One hundred and eighty-five consecutive patients with desmoid tumors treated at our institution between the years 1965 and 1994 were retrospectively reviewed and form the cohort of this analysis. Surgery alone was the treatment for 122 patients, surgery and radiation for 46 (combined modality), and radiation alone for 21. There were 108 women and 81 men. Patients' ages ranged from 1–81 years, with a mean and median of 31 and 29 years, respectively. Size ranged from 1–28 cm, with a median of 8.4 and 7 cm, respectively. One hundred and four patients (55%) presented after one (54 patients) or more (50 patients) local recurrences. Margin status for the surgery alone group was microscopic-positive in 40 (33%), microscopic-negative in 78 (64%), and gross-positive in 4 (3%). Margin status for the combined modality group was microscopic-positive in 33 (72%) and microscopic-negative in 13 (23%). All patients in the radiation alone group were treated for gross disease. Median follow-up was 9.4 years.

*Results*: The 5- and 10-year overall actuarial relapse rate was 30% and 33%, respectively. Uncorrected survival rates were 96%, 92%, and 87% at 5, 10, and 15 years, respectively. For the patients treated with surgery alone the actuarial recurrence rate was 38% at 10 years. The 10 year recurrence rates for the margin-negative and margin-positive patients were 27% and 54% (p=0.003), respectively. For the combined modality patients the 10-year actuarial recurrence rate was 25%. Among 13 margin-negative patients the 10 year recurrence rate was 15%, whereas 33 margin-positive patients had a 10 year recurrence rate of 31% (p=0.5). The difference in recurrence rates for patients with microscopically positive margins treated with surgery alone versus combined modality was significant (p=0.007). On multivariate analysis only age >30 and combined modality treatment (versus surgery alone) independently correlated with a decreaed rate of recurrence. For patients treated with radiation alone the 10-year recurrence rate was 24%.

*Conclusion*: Wide local excision with negative pathologic margins is the treatment of choice for most desmoid tumors. Function sparing resection is appropriate since adjuvant radiation abrogates the adverse impact of positive margins. Unresectable disease should be treated with definitive radiation with the expectation of excellent disease-free survival.

### PRE-OPERATIVE CHEMOTHERAPY AND RADIOTHERAPY (RT) FOR POOR PROGNOSIS EXTREMITY SOFT TISSUE SARCOMAS

***B. Brockstein**, T. Peabody, B. Samuels, A. Mundt, A. Awan, M. Simon*

*The University of Chicaco, Chicago, IL 60637, USA*

Although the majority of soft tissue sarcomas can be treated with limb salvage surgery, a minority of tumors are unresectable and require amputation or resection with a high risk of local recurrence. For this group of patients (pts), pre-surgical therapy with chemotherapy and radiotherapy may allow limb salvage. Additionally, early treatment of micrometastases may reduce the risk of distant failure and subsequent death. We utilized the regimen of Eilber et al. (Proc ASCO, 1994, abstract 1645) to confirm its efficacy, but limited its use to unresectable and marginally resectable pts. From 8/94 until 7/98, a total of 17 pts received 3 cycles of pre-operative chemotherapy, which was repeated, on a case by case basis, approximately 4 weeks post-operatively. RT (350 cGy × 8 fractions in 10 days) was given with (5 pts), before (2 pts), or after (7 pts) the second cycle of chemotherapy. 3 pts received no RT. Chemotherapy consisted of: cycles 1,2,4,5—Ifosfamide 2800 mg/m$^2$/day by 1–2 hour (h) infusion × 5 days; cycle 3,6 doxorubicin 60 mg/m$^2$ by 48 h infusion and cisplatin 120 mg/m$^2$ by 4 h infusion. Demographics included; 9 males (53%) and 8 females (47%); median age—52 (21–77); lower extremity—12 (70%), upper extremity—5 (30%); metastatic disease—2pts (12%), localized disease—15 (88%). Resectability included 7/13 (54%) unresectable, 4/13 (31%) marginally resectable—2/13 (15%) resectable. Number of cycles of chemotherapy received (13 currently evaluable); 2—2 pts, 3—3 pts, 4—3 pts, 5—3 pts, ≥6—2 pts. 14 pts received full RT dose and 3 additionally received a postoperative boost. 3 pts received no RT (2—progression of metastases, 1—amputation due to rapid progression). Major complications of preoperative therapy (13 evaluable currently) included ifosfamide neurocortical toxicity—5 pts, proximal renal tubular acidosis—1 pt, hemorrhagic cystitis—1 pt. Wound healing was delayed in 3/13 pts (23%). Radiologic responses were PR—2, stable—4, progression—3, unevaluable 8. Histological responses were (12 currently evaluable) ≥90 necrosis—6/11 (55%), 50–89% necrosis—2/11 (22%), <50% necrosis—3/11 (33%), 1—no surgery. With a median follow-up of 12 months, there has been 1/13 local recurrence, 2/13 new distant failures, 2/13 with progression of metastatic disease, and 1/13 second primary. 2/13 (15%) pts have died, and 2/13 (15%) have required amputation. Of particular interest, all 3 synovial sarcomas were non-responders. We conclude that in this group of patients with unresectable or difficult to resect disease, aggressive preoperative chemotherapy and RT allowed limb salvage in most patients (85%). We plan to modify the protocol to include additional doxorubicin pre-operatively and post-operatively.

### IMMUNOTHERAPY OF METASTATIC LEIOMYOSARCOMA WITH AUTOLOGOUS TUMOR VACCINE-PRIMED LYMPH NODE LYMPHOCYTES AND INTERLEUKIN-2

***V. K. Sondak**, G. Jiang, R. I. Cross, L. H. Baker, J. J. Mulé, A. E. Chang*

*Multidisciplinary Sarcoma Clinic, University of Michigan Comprehensive Cancer Center, Ann Arbor, MI 48109, USA*

Metastatic leiomyosarcoma, whether it emanates from the gastrointestinal tract, the uterus or other soft tissue or visceral sites, has proven difficult to treat with conventional modalities. New approaches are clearly worth evaluating. Several disparate lines of reasoning support investigations of immunotherapy in this disease: Some forms of leiomyosarcoma are more frequent in immunosuppressed patients and may be associated with Epstein-Barr virus, providing a potential antigenic target. Human T cells are now known to be capable of recognizing antigens on human sarcoma cells. Some patients with metastatic leiomyosarcoma have relatively protracted courses and slowly progressive disease. From May 1997 through July 1998, we entered 8 patients—ages 29 to 70—with metastatic leiomyosarcoma onto an adoptive immunotherapy protocol involving vaccination with irradiated autologous tumor cells plus BCG followed 10 to 14 days later by removal of the regional lymph nodes draining the vaccine sites. These "vaccine-primed" lymph node lymphocytes were sequentially cultured in vitro in anti-CD3 monoclonal antibody (OKT 3) and a low concentration of interleukin 2 (60 Cetus U/ml) for 2 to 3 weeks. Unless tumor progression supervened, activated lymphocytes were administered intravenously followed by moderate-dose interleukin 2 (180,000 Cetus U/kg t.i.d. for 15 doses). Patients manifesting stable or regressing disease were retreated with a second course of interleukin 2. To be eligible, patients had to have accessible or previously cryopreserved tumor and a life expectancy of at least 3 months.

Of the 8 patients, the primary site was gastric in 2, uterine in 2, and small bowel, renal, retroperitoneal and soft tissue in 1 each. All patients had measurable metastatic disease in liver (6 patients) or lung (2 patients) as well as soft tissue sites; no patient had exclusively soft tissue disease. Four patients (1 uterine, 3 gastrointestinal primaries) had received no prior chemotherapy, the remaining patients had all received doxorubicin alone or with ifosfamide. Two patients had rapid progression of tumor and were removed from study prior to the administration of interleukin 2. The remaining 6 patients were all treated with activated lymphocytes plus interleukin 2 and tolerated the therapy well. Dose reductions were required in 2 cases because of renal or hepatic toxicity, no patients required ICU admission or vasopressors. Of the five patients who have been assessed after their initial course of therapy, four met the study conditions for and received a second course of interleukin 2.

Adoptive immunotherapy with autologous tumor vaccine-primed lymph node lymphocytes and interleukin 2 is complex and resource-intensive, but feasible in patients with metastatic leiomyosarcoma. Further investigations of this and other forms of immunotherapy appear to be warranted in this disease.

Acknowledgements: Funded by NIH grants 1R21CA72034 and M01-RR00042.

### IMMUNOGENICITY OF CHROMOSOMAL FUSION PROTEIN SEQUENCES ASSOCIATED WITH SYNOVIAL SARCOMA

***B. S.Worley**, T. J. Goletz, L. J. Helman, J. A. Berzofsky*

*Metabolism Branch and Pediatrics Branch, National Cancer Institute, National Institutes of Health, Bethesda, MD 20892, USA*

Synovial sarcoma (SS) is an aggressive, soft-tissue malignancy occurring primarily in the extremities of adolescents and young adults. More than 70% of SS cases have been attributed to a t(X;18)(p11;q11) chromosomal translocation which fuses the SYT gene from chromosome 18 to either the SSX1 or SSX2 gene on chromosome X. The resulting SYT-SSX1 and SYT-SSX2 fusion proteins are believed to function as aberrant transcriptional regulators. The fusion of SYT to SSX1 or SSX2 creates a unique peptide sequence at the breakpoint which is not expressed in normal cells. Because the t(X;18)(p11;q11) translocation is associated with SS tumor cells, we proposed that the SYT/SSX fusion protein could serve as a tumor-associated antigen. To determine the immunogenicity of the breakpoint peptide sequence, two representative overlapping 17mer peptides (SS1 and SS2, both present in the two types of fusions) were synthesized, both of which span the breakpoint region. Immunization of C57BL/6 mice with SS1-pulsed spleen cells elicited an SS1-specific, $CD8^+$, class I-restricted T cell response, as seen by *in vitro* CTL, activity analysis. Efforts are ongoing to develop an animal model in which to assess the *in vivo* efficacy of the peptides for anti-tumor therapies. Application of this approach in novel immunotherapies depends upon the binding of these peptides to HLA molecules. Therefore, to assess binding of SS1 and SS2 to various HLA molecules, these peptides were tested using T2 cells transfected with HLA molecules and FACS analysis. The SS2 peptide specifically bound both HLA-B7 and HLA-B27 at 100 μM concentration, increasing expression of the class I HLA molecules about 2-fold. The SS1 peptide also specifically bound HLA-B7 at 100 μM concentration, increasing HLA-B7 expression about 3.5-fold. These results suggest that the SYT-SSX1 and SYT-SSX2 fusion proteins potentially encode an antigenic amino acid sequence which specifically binds human HLA molecules. This may be useful in the development of novel immunotherapies for SS.

### PRELIMINARY RESULTS OF A TWO-ARM PHASE 2 TRIAL OF GEMCITABINE IN PATIENTS WITH GASTROINTESTINAL LEIOMYOSARCOMAS AND OTHER SOFT-TISSUE SARCOMAS (STS)

***S. R. Patel**, J. Jenkins, N. E. Papadopoulos, M. A. Burgess, C. Plager, P. W. T Pisters, B. W. Feig, K. Hunt, A. Pollack, G. Zagars, R. E. Pollock, R. S. Benjamin*

*The Sarcoma Center, University of Texas M.D. Anderson Cancer Center, 1515 Holcombe Blvd., Houston, Texas 77030, USA*

A limited number of chemotherapeutic agents have activity in soft-tissue sarcomas. It is therefore important to test newer agents for their activity in this disease. We are currently evaluating Gemcitabine, a nucleoside analog with activity in a variety of solid tumors, in a two-arm phase 2 study. Patients with recurrent/metastatic soft-tissue sarcomas who have received or refused standard chemotherapy with adriamycin and ifosfamide constitute one arm and patients with GI leiomyosarcoma irrespective of prior chemotherapy exposure (due to their know resistance to standard chemotherapy) constitute the other arm. Gemcitabine was given at a dose of 1000 $mg/m^2/wk \times 7$ followed by 1 week off and re-staging to assess response. Patients with responding or stable disease were then continued at a dose of 1000 $mg/m^2/wk \times 3$ followed by 1 week off, and cycles were repeated every 4 weeks until maximum response or progression. A total of 19 patients are evaluable for response, 11 GI leiomyosarcomas and 8 other STS. There were eight males and 11 females and the median age was 53 (28–75) years. All patients had a PS for 0–1. One patient out of 11 GI leio patients has demonstrated a minor response, while two patients out of the 8 other STS have achieved partial responses. One of the responders had a metastatic angiosarcoma to lungs and the other one had lung metastases from a leiomyosarcoma of uterine origin. Treatment was generally very well tolerated. Four patients experienced grade 3 neutropenia and three patients experienced grade 3 thrombocytopenia leading to delays in therapy without any other consequences. Two patients had a grade 3 increase in transaminase levels which was self-limiting. Two patients had grade 3 myalgias. This preliminary data is encouraging and suggestive of activity of Gemcitabine in soft-tissue sarcomas. Our study is continuing to accrue more patients to better define the level of activity.

### A PHASE II STUDY OF RALTITREXED ('TOMUDEX') AS SECOND OR THIRD LINE TREATMENT FOR PATIENTS (PTS) WITH ADVANCED SOFT TISSUE SARCOMAS (ASTS) REFRACTORY TO DOXORUBICIN CONTAINING REGIMENS

***J.-Y. Blay**, I. Judson, S. Rodenhuis, C. Hermans, M. Smith*, M. van Glabbeke, J.Verweij*

*The EORTC Soft Tissue and Bone Sarcoma Group (STBSG), EORTC, 1200 Brussels, Belgium; *Zeneca Ltd, UK*

ASTS refractory to doxorubicin (dox) and ifosfamide (ifo) are highly resistant to chemotherapy. The investigation of new drugs is therefore warranted in these pts. To establish the efficacy and safety of Raltitrexed ('Tomudex') in ASTS refractory to one or two lines of dox and ifo containing regimens, a phase II study was conducted between August 1997 and December 1997 in 8 centers of the EORTC STBSG group. Raltitrexed was given at 3 mg/m2/course as a 15 min IV infusion every 3 weeks. 23 pts were included and 21 are evaluable for toxicity and response. Median age was 54 (range 25–73) with 15 males and 6 females. Performance status was 0 in 9 (43%) pts, 1 in 12 (57%) pts. 16 pts had previously received chemotherapy in metastatic phase, 4 as adjuvant, and 1 both. The primary tumor was located in the trunk (n=11), in the limbs (n=7), in the head and neck (n=3). The predominant histology was leiomyosarcoma (n=7, 33%). Respectively 4, 13, 1, 1, and 2 pts received 1, 2, 4, 5, or 6 courses of Raltitrexed. The best response was stable disease in 4 (19%) pts, and disease progression in 17 pts (81%), with a median time to disease progression of 6 weeks. The treatment was well tolerated with only 1 pt experiencing grade 4 neutropenia and thrombopenia (5%), 1 grade 3 nausea (5%), 1 (5%) lethargy, 1(5%) headache, 1 (5%) asthenia. A single pt (5%) experienced febrile neutropenia. We conclude that Raltitrexed is an ineffective treatment for ASTS failing conventional chemotherapy with dox and ifo. Tomudex is a trademark, a property of Zeneca Ltd.

### FIVE-YEAR SURVIVORS IN PATIENTS (PTS) WITH ADVANCED SOFT TISSUE SARCOMA (ASTS) TREATED WITH DOXORUBICIN: A STUDY ON 1742 PATIENTS (STBSG)

*J.-Y. Blay, M. van Glabbeke, O. S. Nielsen, A. T. van Oosterom, T. Tursz, J. W. Oosterhuis, J. Verweij*

*The EORTC Soft Tissue and Bone Sarcoma Group (STBSG), EORTC, 1200 Brussels, Belgium*

The characteristics of pts with ASTS still alive 5 years (yrs) after initial treatment with doxorubicin (DXR), i.e. long term survivors (LTS), were analyzed among the 1742 pts treated between 1976 and 1990 in trials of EORTC STBSG group. The median overall survival of this series was 11.3 months and the projected 5-years (yrs) survival was 8.6%. 39 pts were alive at 5 yrs among the 1308 uncensored pts. The percentages of females (69% vs 49%), of grade 1 tumors (30% vs 6%), and of pts with an initial performance status (PS) of 0 (49% vs 27%) were superior in the LTS subgroup as compared to other pts ($p<0.01$). Histological subtypes were not significantly different in LTS as compared to other pts. LTS pts less frequently had grade 3 tumors (35% vs 64%), and liver metastasis (5% vs 18%) ($p<0.01$). Despite of these differences, LTS were observed in all categories of pts. A complete response (CR) to DXR was a major parameter correlated to 5-yrs survival: respectively, 18% (11/60) of pts in CR, 4% (9/215) of pts in partial response (PR), 2% (13/482) of pts in stable disease (SD), and 1% (3/372) of pts in progressive disease (PD) after DXR were alive at 5 yrs. Patients in CR, PR, SD and PD represented 31%, 25%, 26%, and 8% of LTS respectively. In multivariate analysis independent parameters correlated to CR and 5-yrs survival were similar to those correlated to overall survival in the same series, i.e. age, liver metastases, PS, grade I tumors. In conclusion, LTS are observed in all prognostic subgroups of pts with ASTS treated with DXR, in particular among pts in CR, in whom 5-yrs survival is 18%. Achievement of CR should be the primary aim of chemotherapy. *This work was supported by the Prix Pierre Bardoux.*

### A NEW PROGNOSTICATION SYSTEM FOR ADULT SOFT TISSUE SARCOMA OF EXTREMITY AND TRUNK WALL, BASED ON TUMOR SIZE, VASCULAR INVASION, AND MICROSCOPIC TUMOR NECROSIS (THE SIN-SYSTEM)

*P. Gustafson, M. Åkerman, T. A. Alvegård, J. M. Coindre, C. D. M. Fletcher, A. Rydholm, H. Willén*

*University Hospital, Lund, Sweden, Institut Bergonié, Bordeaux, France; Brigham and Women's Hospital, Boston, MA, USA*

A clinically useful prognostication system should be reproducible and give good separation between two groups—one with good and one with poor prognosis. We have recently proposed a system based on three negative prognostic factors large tumor size, vascular invasion, and microscopic tumor necrosis. Tumors which exhibit 2 or 3 factors are categorized as high-grade, the others as low-grade. We have now tested this system for reproducibility both as regards classification of necrosis and vascular invasion, and as regards prognostic strength related to grading.

We selected 200 adult patients with STS of the extremity or trunk wall, 100 from the Musculoskeletal Tumor Center, Lund Sweden and 100 from the Institut Bergonié, Bordeaux, France. All patients had been treated by surgery. The median follow-up for the 117 survivors was 10 (1.5–27) years. All slides from all tumors were reviewed independently by three groups of pathologists for the presence or absence of vascular invasion and microscopic tumor necrosis, without knowledge of the clinical course. Tumor size was considered same. The prognostic strength was compared using the grading obtained by the different pathologists. Concordance in classification was assessed by Kappa-analysis. Outcome related to grading was assessed by Kaplan-Meier technique. Concordance in classification of vascular invasion, microscopic tumor necrosis, and grading was seen respectively in 156 (78%), 154 (77%), and 167 (84%) of the 200 tumors. Based on the different observers grading, the cumulative 5-year metastasis-free survival rate in the 200 patients varied for patients with low-grade tumors between 0.85 and 0.80 and for patients with high-grade tumors between 0.48 and 0.43. The kappa-value for grading between all three groups was 0.77.

Classification of vascular invasion and microscopic tumor necrosis seems to have acceptable reproducibility. This so called SIN-system gave similar survival rates when used by different observers and applied to different series of STS patients. It gave good separation between patients into two groups with high or low risk for metastasis.

### CAN WE EXPLAIN THE DIFFERENT PROGNOSTIC FACTORS FOR RESPONSE AND SURVIVAL IN THE VARIOUS HISTOLOGICAL SUBTYPES OF ADVANCED SOFT TISSUE SARCOMA? AN ANALYSIS OF 2185 PATIENTS FROM THE EORTC SOFT TISSUE AND BONE SARCOMA GROUP DATABASE

*M. van Glabbeke, J. W. Oosterhuis, A. T. van Oosterom, O. S. Nielsen, A. Le Cesne, J. Radford, H. Mouridsen, S. Rodenhuis, A. Kirkpatrick, J. Werweij*

*EORTC Data Center, Brussels, Belgium; EORTC STBSG*

This prognostic factors analysis included 2185 patients with advanced soft tissue sarcomas, treated in 7 clinical trials investigating anthracycline containing regimens as 1st line chemotherapy. Overall survival (median: 51 weeks) and response to chemotherapy (26% CR/PR) were the two end-points. Cofactors were sex, age, performance status, prior therapies, presence of locoregional or recurrent disease, lung, liver, and bone metastases at trial entry, delay since initial sarcoma diagnostic, histological type and grade. Both univariate and multivariate analyses were performed.

Multivariate analysis of survival (Cox model) selected the following variables as favorable prognostic factors: good performance status ($P<0.0001$), absence of liver metastases ($P=0.0001$), low histological grade ($P=0.0002$), long delay since initial diagnostic ($P=0.0004$) and young age ($P=0.0045$). Multivariate analysis of response (logistic model) selected the following variables as favorable prognostic factors: absence of liver metastases ($P<0.0001$), young age ($P=0.0024$), high histological grade ($P=0.0051$) and liposarcoma ($P=0.0065$).

The univariate analysis also underlined the prognostic importance of histological subtype. Patients with liposarcoma and synovial sarcoma had a significantly better survival than patients with other cell types, patients with malignant fibrous histiocytoma had a worse survival; liposarcoma patients had a higher response rate and leiomyosarcoma patients had a lower response rate.

Our study confirms that high grade tumors are chemosensitive, despite their poor survival prognosis. As far as histological subtype is concerned, liposarcoma appeared as chemosensitive tumors with a good survival prognosis. These tumors are usually low grade, which explains the favorable survival, but not the observed high response rate. Other histological subtypes dropped out of the multivariate models, but the present analysis explained their prognostic value on the basis of their correlation with other prognostic factors.

We concluded that, for advanced soft tissue sarcoma, response to chemotherapy is not predicted by the same factors as overall survival. This suggests that response should not be the only end-point for evaluation of new agents. Our future plans include the addition of original anatomic site of disease in the model. We are expecting to better characterize the characteristics and prognostic of the different histological subtypes, and will evaluate if phase II trials of new agents should address specific subtypes.

### COMPUTER ASSISTED CYOTGENETIC ANALYSIS OF 53 MALIGNANT PERIPHERAL NERVE SHEATH TUMORS: SPORADIC VERSUS NEUROFIBROMATOSIS TYPE 1 ASSOCIATED MALIGNANT SCHWANNOMAS

***B. E. C Plaat[1], H. J. Hoekstra[2], W. M. Molenaar[1], M. F. Mastik[1], G. te Meerman[3], E. van den Berg[3]***

*Depts of Pathology[1], Surgical Oncology[2], Medical Genetics[3], University Hospital Groningen, PO Box 30.001, 9700 RB Groningen, The Netherlands*

*Introduction*: Malignant peripheral nerve sheath tumors (MPNSTs) are a relatively rare type of soft tissue sarcoma. Half of the patients suffer from von Recklinghausen's neurofibromatosis (NF-1). Cytogenetic studies in small groups of patients revealed complex karyotypes with no consistent changes. A computer assisted cytogenetic analysis was used to determine recurrent cytogenetic alterations in MPNSTs and to allow direct cytogenetic comparison between NF-1 associated and sporadic MPNSTs.

*Material and methods*: Karyotypes of 53 MPNSTs (46 from the literature and 7 new cases) were interpreted and the gains and losses of chromosomal material in 86 ISCN-described chromosomal regions as well as the breakpoints were entered into a database. To detect significant gains or losses of chromosomal material, 95%-confidence intervals were constructed. Differences between NF-1 associated and sporadic MPNSTs were studied by the creation of karyographs and statistical analysis.

*Results*: Significant ($p<0.05$) loss was observed in 9p2, 11p1, 11q2 and 18p1. Gain of chromosomal material was found in chromosome 7, especially 7q1 ($p<0.05$). Most involved breakpoints were: 1p13, 1q21, 7p22, 9p11, 17p11, 17q11, 22q11. Differences between NF-1 associated and sporadic MPNSTs included a relative loss of chromosomal material in NF-1 associated MPNSTs in 1p3, 4p1 and 21p1-q2 as well as a relative gain in 15p1-q1. Differences in breakpoints were observed in 1p21-22 (28% of NF-1 vs 0% of sporadic MPNSTs), 1p32-34 (17% vs 0%), 8p11–12 (7% vs 27%) and 17q10–12 (24% vs 7%).

*Conclusion*: This computer assisted approach shows that losses in 9p2 and gains in 7q1 could be of oncogenetic importance in MPNSTs. Loss of 17q1, on which the NF-1 gene has been located (17q11.2), is not a common cytogenetic finding in NF-1 associated MPNSTs. The observed differences between NF-1 associated and sporadic MPNSTs might reflect different oncogenetic pathways.

### P-GLYCOPROTEIN AS A PREDICTOR OF OUTCOME IN OSTEOSARCOMA

***F. J. Hornicek, M. C. Gebhardt, M. W. Wolfe, F. D. Kharrazi, H. Takeshita, H. J. Mankin***

*Orthopaedic Oncology Unit, Massachusetts General Hospital and Children's Hospital, Harvard Medical School, Boston, MA 02114, USA*

*Purpose*: To establish a relationship between the expression of P-glycoprotein by osteosarcomas and the rate of metastasis and death.

*Materials and methods*: A retrospective review of 172 osteosarcoma patients diagnosed between 1987 and 1992 was performed. Forty patients had p-glycoprotein levels available. The majority of the osteosarcomas were stage II-B (33 patients), with the remaining seven being stage III. Tumor sites included 25 femora, 7 humeri, 5 tibiae, and one each of pelvis, radius and fibula. The expression of P-glycoprotein by cultured tumor cells from biopsy specimens was determined using immunofluorescent microscopy.

*Results*: More patients with detectable P-glycoprotein (12/18 or 67%) developed metastases as compared to those patients with undetectable P-glycoprotein (9/22 or 41%; N.S.). Similarly, ten of 18 patients (56%) with tumors expressing P-glycoprotein died of disease, while only 4 of 22 (18%) with no detectable P-glycoprotein died (chi-square; $p<0.02$). Among the patients with stage III disease at presentation, two of three with P-glycoprotein expression died, while only one of four without P-glycoprotein died.

*Conclusion*: Expression of P-glycoprotein by tumor cells appears to be associated with an increased incidence of death in stage II and III osteosarcoma.

### EVALUATION OF EWING'S SARCOMA INFORMATION ON THE INTERNET

***G. J. Golladay**, J. S. Biermann, M. L. Greenfield, L. H. Baker*

*University of Michigan Comprehensive Cancer Center, Ann Arbor, MI 48109, USA*

*Background*: The Internet is a rapidly expanding and widely used information source. Although many people are using the Internet to search for medical information, there are no guarantees that information retrieved is pertinent or correct. We chose Ewing's sarcoma as a search item to evaluate the accuracy and relevance of Internet medical search results.

*Methods*: We developed a guide to evaluate all medical information that might appear on Web pages, but limited our study to a search of Ewing's Sarcoma. The guide demonstrated good interobserver and intraobserver reliability during preliminary and final study testing. We evaluated the first 100 URLs retrieved using four search engines searching the phrase, "Ewing's Sarcoma". We evaluated each Web page for type of medical information presented, references, the presence of peer review, and accuracy.

*Results*: Of 400 search-engine generated URLs, there were 29 duplications. Only 170 Web pages contained medical information relevant to our search term. 34.5% of Web pages contained no reference to peer review. Six percent of non-peer reviewed Web pages contained factually incorrect information.

*Conclusions*: We developed a reliable rating instrument to evaluate medical information on the Internet for relevance to the search term, type of medical information presented, and presence of peer review. We demonstrated that searching for medical information regarding Ewing's sarcoma in the Internet yielded both peer reviewed and non-peer reviewed information and found that factual inaccuracies in the non-peer reviewed material were present, at a rate of six percent. Although this number is relatively small, some of the inaccuracies identified had significant potential to lead to serious harm to patients with Ewing's sarcoma.

### PATIENT USE OF THE INTERNET FOR MEDICAL INFORMATION IN AN OUTPATIENT CANCER CENTER

***G. J. Golladay**, M. L. Greenfield, L. H. Baker, J. S. Biermann*

*University of Michigan Comprehensive Cancer Center, Ann Arbor, MI 48109, USA*

*Background*: While the Internet is a growing source of medical information for patients as well as health care providers the extent to which patients are using the Internet to gain information related to their diagnosis or treatment or both remains obscure. To assess patient use we studied a sample of comprehensive cancer center patients after designing and field testing a specific questionnaire.

*Methods*: All patients presenting to the outpatient clinics of two faculty (JSB and LHB) during a three week period were administered a confidential questionnaire. The questionnaire was designed to measure the use of the Internet, the search method by which information was retrieved, who obtained the information, and the attitude of the patient towards the new information obtained. Patients were also asked to respond to the same questionnaire by mail at a later date in order to assess the reliability of the instrument.

*Results*: 200 patients participated. 87 of the surveyed patients (43.5%) used the Internet to search for medical information. 43 patients used the Internet themselves while 78 someone else to search the Internet for them (including 34 of the primary users). 37 of the users would recommend the Internet as an information resource for other patients becuse of the speed and ease of information retrieval the Internet offers. 10 patients expressed concern about the large volume and credibility of information accessible on the Internet. Sufficient patients volunteered to repeat the use of the questionnaire to assure adequate reliability.

*Conclusions*: Nearly half of surveyed patients in this sample had obtained Internet information related to their diagnosis, prognosis, or treatment. The Internet is a rapidly developing medical information resource for patients that requires physician input for optimal patient benefit. To this end, our Cancer Center has a patient education resource center that assists in teaching patients and families how to do searches and we maintain a committee of health care providers who recommend Web sites that are deemed accurate and useful.

### THALLIUM UPTAKE IN CHONDROID LESIONS

***K. T. Templeton**, T. G. Raveill, O. Tawfik*

*University of Kansas Medical Center, Kansas City, Kansas 66160, USA*

Heterogeneity within cartilagenous lesions can make grading and surgical planning difficult. In conjunction with standard imaging modalities, $^{201}$Thallium has been utilized in the evaluation of bone and soft tissue sarcomas, with 100 percent uptake noted in primary and recurrent sarcomas. It has also been shown to have an accuracy rate of 97 percent in determining benign vs. malignant bone and soft tissue lesions, with high-grade sarcomas occasionally demonstrating a "doughnut" sign. In chondroid lesions, T1201 has been shown to differentiate intermediate/high from low-grade/benign lesions.

T1201 imaging was utilized in three patients with radiographic evidence of chondroid lesions which were read as consistent with low-grade chondrosarcoma on open biopsy. T1201 scans were obtained to evaluate for the presence and location of higher grade areas within the lesions. All patients received 5mCi of T1201 with early (20 minute) and, in two, delayed (3 hour) imaging. Both lesions were also scanned with SPECT. After resection, histological mapping of the tumors was performed to compare with T1201 images.

The areas noted on histological exam to be low-to-intermediate grade demonstrated a T1201 uptake ratio (compared to the contra lateral normal site) of 0.99 to 1.11. This was unchanged on delayed images (ratios 1.06–

1.12). In comparison, the higher grade areas had uptake of 1.26–1.64 cts/pixel (or 1.38–1.47 max cts) of the contra lateral normal site. In addition, four other patients with chondroid lesions, diagnosed radiographically, have been followed with periodic clinical exam and roentgenograms. All had T1201 scans with uptake noted to be equivalent to the contra lateral site. All of these patients remain asymptomatic with no change in their radiographs during follow-up (range 5 mos.–4 yrs.)

T1201 uptake, mediated through the Na-K ATPase dependent pump, is dependent not only on blood flow but also tumor of cell viability and activity. With a degree of heterogeneity seen in chondroid lesions, T1201 may help illucidate those tumors, and regions within tumors, that are more aggressive and therefore need more aggressive management despite a relatively low grade as seen on open biopsy.

### CHEMOTHERAPY, IRRADIATION, AND SURGERY FOR FUNCTION-PRESERVING CURATIVE THERAPY OF PRIMARY EXTREMITY SOFT TISSUE SARCOMAS: INITIAL TREATMENT WITH I-MAP + GM-CSF—PRELIMINARY REPORT

*J. H. Edmonson, I. A. Petersen, T. C. Shives, M. G. Rock, M. G. Haddock, F. H. Sim, W. J. Maples, M. I. O'Connor, L. L. Gunderson, M. L. Foo, D. J. Pritchard, J. C. Buckner, S. L. Stafford, M. R. Mahoney*

*Mayo Clinic, Rochester, MN 55905, USA*

Between 3/94 and 10/97 20 women and 19 men, median age 51 yrs (27–76 yrs), were treated for cure of their high grade primary extremity and limb girdle soft tissue sarcomas with preoperative chemotherapy and irradiation. This involved two monthly cycles of IMAP (ifosfamide 2,500 mg/m$^2$ + MESNA 2,500 mg/m$^2$ daily for two days with mitomycin 4 /mg/m$^2$, doxorubicin 40 mg/m$^2$, and cisplatin 60 mg/m$^2$ added on the second day). Beginning six days before chemotherapy each month they received four days of GM-CSF (sargramostim) 250 mcg/m$^2$ S.C. every 12 hours. This was then continued for 14 more days the day after chemotherapy was finished each month. External beam irradiation was begun at month three and continued five days weekly for approximately 4,500–5,000 cGy in five weeks, accompanied by adjuvant doses of mitomycin 6 mg/m$^2$, doxorubicin 30 mg/m$^2$, and cisplatin 45 mg/m$^2$ given thrice (before, midway, and after irradiation.) Definitive surgery accompanied by intraoperative electron irradiation or brachytherapy and/or further external beam treatment (to a total dose of 5,500–7,000 cGy) was scheduled to occur after a one month rest interval. No postoperative chemotherapy was given. Sites of origin were thigh 23, pelvic girdle 5, leg 4, arm 3, shoulder girdle 2, and forearm 2. Maximal tumor diameters ranged from 1.6 to 30 cm (median 10 cm). Histologic types were malignant fibrous histiocytoma 20, synovial sarcoma 5, extraosseous osteosarcoma 4, malignant peripheral nerve sheath tumor 3, spindle cell 2, fibrosarcoma 2, and leiomyosarcoma 3. Surgery involved radical excision in two patients, wide excision in 22 patients (1 contaminated), marginal excision in 14 (1 contaminated), and no surgical excision in one patient (who developed brain metastasis during preoperative treatment). Significant complications included tissue necrosis requiring debridement and secondary closure (1), intralesional hemorrhage and necrosis requiring amputation (1), and pathologic fractures with infection requiring amputation (1). Limbs remained intact in 37 of the 39 patients. The prescribed irradiation was received in 36 cases at Mayo Clinic; two patients received preoperative irradiation elsewhere; and one patient (who experienced massive intralesional hemorrhage and amputation) received none. Thirty four patients received two cycles of IMAP chemotherapy and five received one cycle. Only eight patients received the full three cycles of MAP chemotherapy concomitant with preoperative irradiation; 12 patients received two cycles; 14 patients received one cycle; and 5 patients received none. With median follow up of 33 months (8–51 mo.) four patients have died of metastatic disease and a fifth died N.E.D. in a motorcycle accident. Two patients have experienced only local recurrence and a total of eight have experienced only metastasis (including the four who died).

Maturing Kaplan-Meier curves suggest favorable survival and time to metastasis experience with this regimen.

### WE DO NEED TO CONFIRM THE RESULTS OF SMALL CHEMOTHERAPY TRIALS IN ADVANCED SOFT TISSUE SARCOMA WITH RANDOMIZED STUDIES: THE EXPERIENCE OF THE EORTC SOFT TISSUE AND BONE SARCOMA GROUP (STBSG)

*M. Van Glabbeke, A. van Oosterom, A. Azzarelli, A. Le Cesne, H. Mouridsen, J. Radford, S. Rodenhuis, J. W. Oosterhuis, C. Hermans, J. Verweij*

*EORTC Data Center and STBSG, Brussels, Belgium*

For the last 20 years, the (STBSG) has conducted successive clinical trials testing new agents and combinations in patients with non pretreated advanced soft tissue sarcoma. Initial results are generally confirmed in large phase III randomized trials. The present work reviews the usefulness of this procedure, and compares the results of the confirmation trials with those of the initial trials.

In 1982, a randomized phase II trial of Ifosfamide (IFOS) vs Cyclophospamide showed a response rate of 25% in 38 patients treated with IFOS (5 g/m2, 24 hr infusion, q 3 wks). In 1992, the same regimen used as the control arm in a randomized phase II trial showed 5% responses in 49 patients.

The original promising IFOS results encouraged the group to test, in 1984, IFOS 5 g/m2 combined with Doxorubicine (DOX) 50 mg/m2. In an initial non randomized study, a response rate of 34% was obtained in 203 patients. On this basis, the group embarked in 1985 on a randomized phase III trial, comparing this combination to DOX alone (75 mg/m2); this study did not show any significant advantage of the IFOS-DOX combination, where 28% responses were observed in 297 patients.

The next step was to increase the dose of DOX to 75 mg/m2 in the combination, which was made possible by the addition of growth factors. A pilot study with IFOS 5 g/m2, DOX 75 mg/m2 and GmCSF conducted in 1988 showed a response rate of 46% in 111 patients; in 1992, the group started a randomized trial comparing this regimen to the previous "standard dose" regimen. Response rates were not statistically significant, and substantially lower than those observed in the initial non randomized trials: 21% in the standard dose regimen and

23% in the high dose regimen (to be compared with the 34% and 46% of the initial pilot studies). Despite these large differences in response rates, no major differences were observed in survival between all these trials.

These results underline the danger of historical control and small size studies in this disease. The profile of patients referred to first line chemotherapy had probably changed over time, and the number of histological subtypes may be responsible for large heterogeneity in recruited patients. Randomization and stratification for known prognostic factors does overcome these problems. Response rate is probably not a valid end-point for comparing chemotherapy regimen in this disease. External review of response which is now a standard practice of the group may partially explain the decrease in response rates observed in our trials.

